# Machine learning approach to integrated endometrial transcriptomic datasets reveals biomarkers predicting uterine receptivity in cattle at seven days after estrous

**DOI:** 10.1038/s41598-020-72988-3

**Published:** 2020-10-12

**Authors:** Maria B. Rabaglino, Haja N. Kadarmideen

**Affiliations:** Quantitative Genetics, Bioinformatics and Computational Biology Group, Department of Applied Mathematics and Computer Science, Technical University of Denmark, Richard Petersens Plads, Building 324, 2800 Kgs. Lyngby, Denmark

**Keywords:** Computational biology and bioinformatics, Physiology, Biomarkers

## Abstract

The main goal was to apply machine learning (ML) methods on integrated multi-transcriptomic data, to identify endometrial genes capable of predicting uterine receptivity according to their expression patterns in the cow. Public data from five studies were re-analyzed. In all of them, endometrial samples were obtained at day 6–7 of the estrous cycle, from cows or heifers of four different European breeds, classified as pregnant (n = 26) or not (n = 26). First, gene selection was performed through supervised and unsupervised ML algorithms. Then, the predictive ability of potential key genes was evaluated through support vector machine as classifier, using the expression levels of the samples from all the breeds but one, to train the model, and the samples from that one breed, to test it. Finally, the biological meaning of the key genes was explored. Fifty genes were identified, and they could predict uterine receptivity with an overall 96.1% accuracy, despite the animal’s breed and category. Genes with higher expression in the pregnant cows were related to circadian rhythm, Wnt receptor signaling pathway, and embryonic development. This novel and robust combination of computational tools allowed the identification of a group of biologically relevant endometrial genes that could support pregnancy in the cattle.

## Introduction

Various bioinformatics and systems biology tools in animal production and health sciences^1^, and specifically in cattle artificial reproduction^2^, focus on integrating biological data layers (genomics and transcriptomics) and application of statistical-bioinformatics methods (e.g. eQTL mapping) to identify functionally relevant targets and biomarkers. However, the emergence of machine learning (ML), as a big data science tool, is less explored in livestock functional genomics in general, and bovine species in particular. ML refers to the use of self-learning algorithms to make sense of big data and is a branch of artificial intelligence that holds great potential for pattern recognition in complex datasets^3,4^, such as the ones derived from the “omics” technologies. In transcriptomic data (captured by either microarrays or RNA-sequencing platforms), expression pattern analysis is central to find functionally relevant groups of genes under different treatment conditions or phenotypic categories. Thus, application of ML tools represents a powerful analytical approach that can be strengthened when it is applied to data integrated from several datasets (i.e., multi-transcriptomic data), which provides a robust overview of the system under study^5^.

Here, we investigated ML methods across multi-transcriptomic data, in the context of characterizing the receptive endometrium at the time of embryo transfer (ET) in European cattle. The endometrial transcriptomic profile should determine a favorable environment for contact and communication with the embryo at around 5–6 days of pregnancy, when the conceptus reaches the uterus^6^. Thus, identification of a receptive endometrium becomes crucial at around 7 days of the estrous cycle, when an embryo is deposited into the uterus after the application of assisted reproductive technologies. Previous works, including ours^7^, have demonstrated that the endometrial transcriptomic profile at day 6 or 7 differs between animals that become pregnant or not. This fact has been shown in Nellore cows after artificial insemination (AI)^8^, Simmental heifers after in vivo produced ET^9,10^, dairy cows after in-vitro produced ET^7^, and cross-breed heifers after AI^11^. These studies have applied high-throughput technologies with the goal of determining the differentially expressed genes in the endometria of animals that resulted pregnant compared to those that do not. Their results helped to shed light on the identification of those genes whose expressions control the fate of uterine receptivity. Nevertheless, results from these studies are not entirely consistent, since several other factors, such as breed and category, can influence gene expression. Therefore, the identification of endometrial genes as biomarkers of receptivity is still challenging.

In the present study, we combined data integration with supervised and unsupervised ML tools to provide actionable knowledge from various endometrial transcriptomic datasets. We hypothesized that such novel computational approach could reveal the main gene expression signature of the receptive endometrium at the time of ET in cattle. The goal of this study was to conduct multi-transcriptomic data integration from five publicly available datasets, including our recent study on endometrial transcriptomics^7^, and apply ML tools to such integrated data, to identify biomarker genes determining uterine receptivity according to their expression patterns. These studies, listed in Table 1, have in common that endometrial samples from European or Taurine cattle *(Bos taurus taurus)* were obtained at day 6–7 of the estrous cycle, and they were classified as receptive (R, n = 26) or not (nonR, n = 26) after ET or AI, depending on the study. Selection and validation of the potential biomarkers were done in three steps involving supervised and unsupervised ML methods. Once these key genes were determined, the final aim was to explore their biological characteristics through predictions in external data to discern the role of estradiol and progesterone in their expression, and network analysis to reveal related genes.

**Table 1:** Characteristics of each dataset selected for data integration and analysis.

Accession number in GEO	Animal breed	Animal category	Method to induce pregnancy	Method of sample collection	Number of samples	Platform for transcriptomic determination	Authors & date of data publication
GSE115756	Holstein	Lactating cows (1st to 3rd lactation)	IVP-ET	Biopsy instrument	R = 8 nonR = 9	Illumina HiSeq 2500 (RNA- sequencing)	Mazzoni et al.^7^—Dec 31, 2019
GSE107741	Japanese Black	Cows	AI / IV-ET	Biopsy instrument	R = 6 nonR = 5	Agilent-023647 B. taurus Oligo Microarray v2	Matsuyama et al. *(article not published)*—Jan 11, 2018
GSE29853	Charolais × Limousine	Heifers	AI	Postmortem peeling from the uterine myometrium	R = 6 nonR = 6	Affymetrix Bovine Genome Array	Killen et al.^11^—Jun 10, 2011
GSE36080	Simmental	Heifers	IV-ET	Cytobrush	R = 3 nonR = 3		Ponsuksili and Wimmers^9^—Jan 01, 2013
GSE20974					R = 3 nonR = 3		Salilew-Wondim et al.^55^—Mar 25, 2010

For all the experiments, endometrial samples from *Bos taurus* cattle were obtained around day 7 of the estrous cycle, and they were classified retrospectively or prospectively according to the pregnancy results. IVP-ET: transfer of in-vitro produced embryos; IV-ET: transfer in-vivo produced embryos; AI: artificial insemination; R and nonR: animals classified as receptive or not, respectively.

## Results

In what follows below, the main results are categorized into four topics.

### Identification of groups of potential biomarker genes through supervised ML

The software BioDiscML^12^ was employed for selection of potential biomarker genes. This software automates the main steps in ML by implementing methods for features and model selection, in order to identify the best model for data classification. The software generated 2097 models, from which only five models presented accuracy higher than 90% in the test set and in the evaluation procedures in the train set. These models were:

- Two models of Bayes Network optimized by accuracy of prediction, which selected 100 and 75 genes
- Two multinomial logistic regression models, also optimized by accuracy of prediction, which selected the same 100 and 75 genes, and
- Bayes Network optimized by False Discovery Rate, which selected 50 genes.

The 100, 75 and 50 selected genes were overlapping, meaning that the 50 genes were repeated in the three groups.

### Identification of the best group of biomarker genes through unsupervised ML

The groups of genes identified as potential biomarkers were evaluated for their ability to blindly cluster apart the R and nonR samples, according to their expression levels, in a hierarchical clustering. As expected, the group of 50 genes showed the best performance (92.3% accuracy). Only one nonR sample from the Holstein cows (nonR_Hols_6) was clustered with the R samples, while three R samples were grouped with the nonR samples. These samples were one from Holstein (R_Hols_3), one from the Charolais x Limousine (R_Cont_3) and one from the second study with the Simmental heifers (R_Sim2_1). The corresponding dendrograms and heat maps for the expression signatures are shown in Supplementary Fig. 1, together with the confusion matrix and accuracy for each classification.

In addition, hierarchical clustering of the genes according to their expression showed two main clusters of genes, corresponding to those with increased or decreased expression in the R cows (up-regulated or down-regulated, respectively).

Table 2 lists the 50 genes, with the respective indication if they were more (UP, n = 32) or less (DOWN, n = 18) expressed in the 7-day endometria of the R animals.

**Table 2:** List of the 50 endometrial genes identified as biomarkers to determine pregnancy status around day 7 of the estrous cycle in the *Bos taurus* cattle.

Ensembl gene ID	Gene symbol	Gene name	Direction
ENSBTAG00000001069	TP53	Tumor protein p53	UP
ENSBTAG00000001568	PPIC	Peptidylprolyl isomerase C	UP
ENSBTAG00000002108	YWHAQ	Tyrosine 3-monooxygenase/tryptophan 5-monooxygenase activation protein theta	UP
ENSBTAG00000002130	SMPD4	Sphingomyelin phosphodiesterase 4	UP
ENSBTAG00000003397	CTBP2	C-terminal binding protein 2	DOWN
ENSBTAG00000003532	TLE4	Transducin like enhancer of split 4	UP
ENSBTAG00000003718	HACL1	2-hydroxyacyl-CoA lyase 1	DOWN
ENSBTAG00000003843	SMARCAL1	SWI/SNF related, matrix associated, actin dependent regulator of chromatin, subfamily a like 1	UP
ENSBTAG00000004459	TMEM45A	Transmembrane protein 45A	UP
ENSBTAG00000004769	NEIL2	Nei like DNA glycosylase 2	DOWN
ENSBTAG00000005092	ROR2	Receptor tyrosine kinase like orphan receptor 2	UP
ENSBTAG00000005462	FXR2	FMR1 autosomal homolog 2	DOWN
ENSBTAG00000006002	DIDO1	Death inducer-obliterator 1	UP
ENSBTAG00000007007	WDR20	WD repeat domain 20	DOWN
ENSBTAG00000008083	SEL1L	SEL1L ERAD E3 ligase adaptor subunit	UP
ENSBTAG00000008181	CHAF1A	Chromatin assembly factor 1 subunit A	UP
ENSBTAG00000008943	ZSCAN12	Zinc finger and SCAN domain containing 12	DOWN
ENSBTAG00000009121	STAG2	Stromal antigen 2	DOWN
ENSBTAG00000009541	SUCLG2	Succinate-CoA ligase GDP-forming beta subunit	DOWN
ENSBTAG00000009863	BHLHE40	Basic helix-loop-helix family member e40	UP
ENSBTAG00000010416	RIN3	Ras and Rab interactor 3	UP
ENSBTAG00000011205	PPP1R42	Protein phosphatase 1 regulatory subunit 42	UP
ENSBTAG00000011818	COL26A1	Collagen type XXVI alpha 1 chain	UP
ENSBTAG00000012454	SLC35A3	Solute carrier family 35 member A3	DOWN
ENSBTAG00000014217	HHEX	Hematopoietically expressed homeobox	UP
ENSBTAG00000014393	CLK2	CDC like kinase 2	UP
ENSBTAG00000014644	DUS2	Dihydrouridine synthase 2	UP
ENSBTAG00000014713	RARRES1	Retinoic acid receptor responder 1	UP
ENSBTAG00000014838	VPS26B	VPS26, retromer complex component B	DOWN
ENSBTAG00000015390	FN3KRP	Fructosamine 3 kinase related protein	DOWN
ENSBTAG00000016977	FUNDC2	FUN14 domain containing 2	UP
ENSBTAG00000017505	PAXIP1	PAX interacting protein 1	UP
ENSBTAG00000017833	RNF19A	Ring finger protein 19A, RBR E3 ubiquitin protein ligase	DOWN
ENSBTAG00000019155	FRS2	Fibroblast growth factor receptor substrate 2	DOWN
ENSBTAG00000020611	GLB1L	Galactosidase beta 1 like	UP
ENSBTAG00000020943	CTU2	Cytosolic thiouridylase subunit 2	DOWN
ENSBTAG00000021151	MYH10	Myosin heavy chain 10	UP
ENSBTAG00000021680	SKA2	Spindle and kinetochore associated complex subunit 2	UP
ENSBTAG00000021768	CCNG2	Cyclin G2	DOWN
ENSBTAG00000023179	TRIB1	Tribbles pseudokinase 1	UP
ENSBTAG00000024240	ACADM	acyl-CoA dehydrogenase, C-4 to C-12 straight chain	DOWN
ENSBTAG00000026290	PIK3C3	Phosphatidylinositol 3-kinase catalytic subunit type 3	DOWN
ENSBTAG00000031385	RFWD2	Ring finger and WD repeat domain 2	UP
ENSBTAG00000032613	SCG5	Secretogranin V	UP
ENSBTAG00000034693	SYT1	Synaptotagmin 1	UP
ENSBTAG00000037757	EBF4	Early B-cell factor 4	UP
ENSBTAG00000038866	UBE2I	Ubiquitin conjugating enzyme E2 I	UP
ENSBTAG00000039980	CLECL1	C-type lectin-like 1	UP
ENSBTAG00000043964	ARL5B	ADP ribosylation factor like GTPase 5B	DOWN
ENSBTAG00000045550	TSPAN6	Tetraspanin 6	UP

The column named “direction” indicates if the gene was more (UP) or less (DOWN) expressed in the endometria of the animals that resulted pregnant.

### Validation of the selected set of biomarker genes through supervised ML

The next step was to verify if the expression signature of the 50 selected genes were able to predict uterine receptivity. For this, we applied Support vector machines (SVM) as classifier, using all the samples but the samples from a given breed as training set, and the samples from such breed as testing set. Therefore, we could discern if the expression signature of these genes would be able to predict uterine receptivity across all the bovine breeds.

The evaluation metrics associated with the confusion matrix for each of the four train/test set are depicted in Table 3. Using the expression of the 50 genes in all the samples, but the samples for a particular breed, to train the SVM, the accuracy to predict correctly the uterine receptivity in that particular breed was 100% for the Japanese and Simmental breeds, 94.1% for the Holstein cows and 91.7% for Charolais x Limousine heifers. One nonR sample from the Holstein cows (non_R_6) was misclassified as R, while one R sample from the Charolais x Limousine heifers was misclassified as nonR (R_Cont_1). Therefore, the overall accuracy was 96.1%.

**Table 3:** Evaluation metrics corresponding to the classifications on pregnancy status based on the expression of the 50 endometrial genes for each breed, using Support Vector Machine as classifier, trained with all the samples except for the samples of the particular breed.

Metric	Holstein	Charolais × Limousine	Japanese black	Simmental
Accuracy	0.941	0.917	1.000	1.000
Kappa	0.883	0.833	1.000	1.000
Accuracy Lower	0.713	0.615	0.715	0.735
Accuracy Upper	0.999	0.998	1.000	1.000
Accuracy Null	0.529	0.500	0.545	0.500
Accuracy PValue	0.000	0.003	0.001	0.000
McNemar PValue	1.000	1.000	NA	NA
Sensitivity	0.889	1.000	1.000	1.000
Specificity	1.000	0.833	1.000	1.000
Positive Predictive Value	1.000	0.857	1.000	1.000
Negative Predictive Value	0.889	1.000	1.000	1.000
Precision	1.000	0.857	1.000	1.000
Recall	0.889	1.000	1.000	1.000
F1	0.941	0.923	1.000	1.000
Prevalence	0.529	0.500	0.455	0.500
Detection Rate	0.471	0.500	0.455	0.500
Detection Prevalence	0.471	0.583	0.455	0.500
Balanced Accuracy	0.944	0.917	1.000	1.000

### Determination of the biological significance of the selected biomarker genes

As a final step, we investigated the biological meaning of the 50 genes through two methods: predictions in external datasets and functional/network analysis.

*Predictions in external datasets:* with the aim of understanding the role of estradiol and progesterone in the expression of the biomarker genes, two datasets were selected to generate the test sets based on the endometrial expression of the 50 biomarker genes. The training set consisted on the expression of the 50 genes in the 52 samples described in Table 1. Predictions of ‘receptive’ samples for each test set were as follow:

Test set 1) Five out of five pregnant heifers with normal progesterone levels (PN), but only one out of five pregnant heifers treated with a progesterone device on day 3 of the estrous cycle (PH).

Test set 2) Three out of three ovariectomized cows receiving a progesterone treatment for six days plus estradiol at day 6 (E2 + P4) but none out of three receiving only the progesterone treatment (P4).

**Figure 1:**
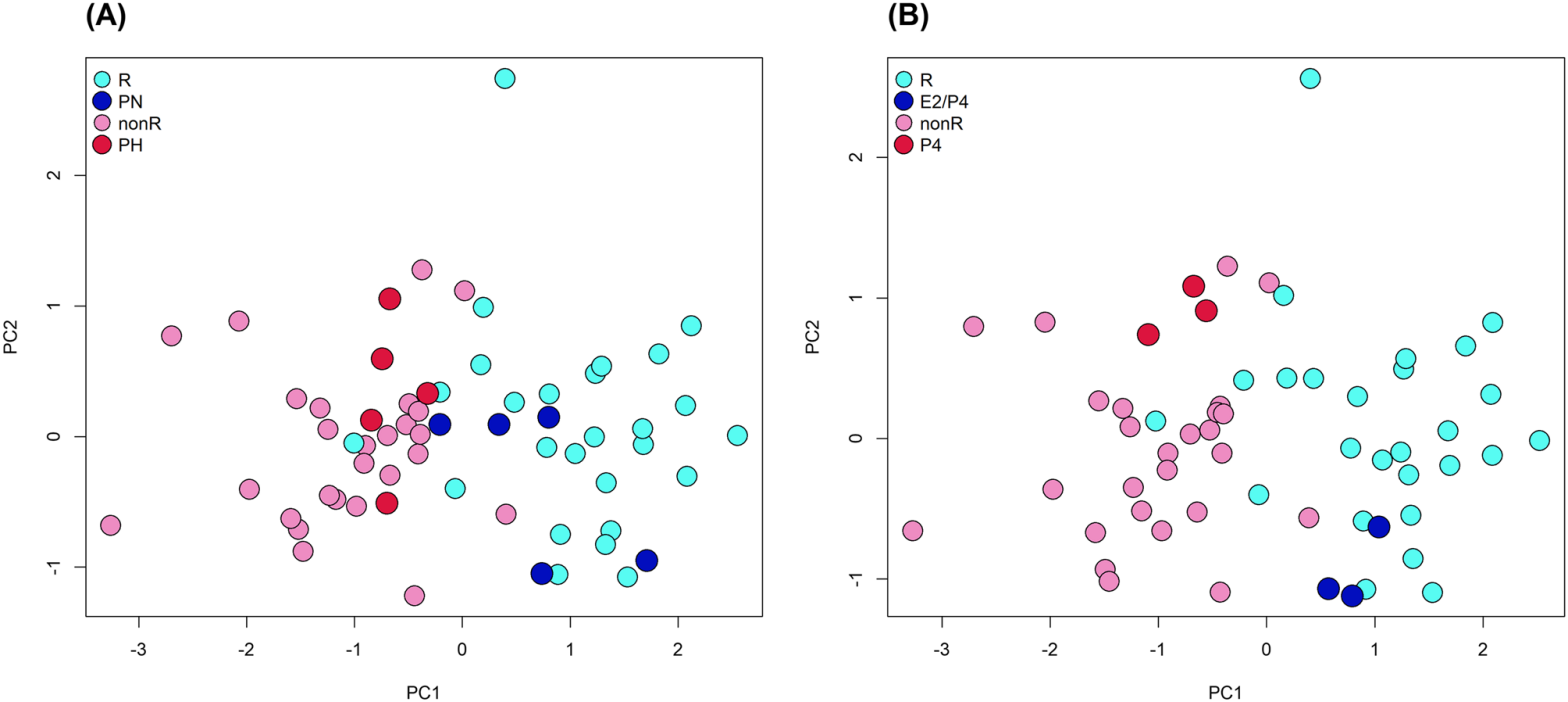
Principal component analysis of samples’ distribution according to the expression of the 50 biomarker genes. The plots show the distribution of samples collected from receptive (R) and non-receptive (nonR) cows, and samples obtained from: (**A**) pregnant cows but treated with a progesterone device from day 3 (PH) or with normal progesterone levels (PN); (**B**) ovariectomized cows receiving a progesterone treatment for 6 days plus estradiol at day 6 (E2 + P4) or only the progesterone treatment (P4).

Accordingly, samples that were classified as ‘receptive’ (PN and E2 + P4) tended to cluster with the R samples, and vice versa for samples classified as ‘non receptive’ (PH and P4), in a PCA plot (Fig. 1).

*Functional/network analysis:* functional classification of the protein class for each gene was performed with the Panther database^13^ and network analysis was done with the Cytoscape software (V. 3.7.2)^14^.

From the 50 genes, 26 genes were classifying into protein classes, from which the most abundant protein class was gene-specific transcriptional regulator (six out of the 26). These regulators were the following transcription factors: Cellular tumor antigen p53 (TP53), Basic helix-loop-helix family member e40 (BHLHE40), Hematopoietically expressed homeobox (HHEX) and Zinc finger and SCAN domain containing 12 (ZSCAN12). The following regulators are transcription co-factors: Transducin like enhancer of split (TLE4) and C-terminal-binding protein 2 (CTBP2). All the genes were more expressed in the animals that become pregnant, except for ZSCAN12 and CTBP2.

The top 100 related genes to the up- and down-regulated biomarker genes in the R animals were inferred and analyzed with Cytoscape (Supplementary Table 1). These genes generated highly connected networks (Supplementary Fig. 2). The significantly enriched biological processes (adj. *p* < 0.05) related to these networks are listed in Supplementary Table 2. The main significant biological processes determined in the network derived from the up-regulated genes in the endometria of the R cows are: positive regulation of biological/cellular process, regulation of gene expression, circadian rhythm, regulation of apoptosis, Wnt receptor signaling pathway, and embryonic development. For the down-regulated genes, the main non-redundant biological processes are: chromosome segregation, lipid modification, negative regulation of biological/cellular process, M phase of cell cycle, and fatty acid oxidation.

## Discussion

So far, most of the studies of the bovine endometrial transcriptome during the early-luteal phase period have utilized bioinformatics methods to detect differentially expressed genes between the groups in that particular study^7–10,15^. The outputs of these investigations are deposited in the public GEO database, which enables the access to a large amount of high-throughput data^16^. Integration of several datasets could lead to a better characterization of the system under study^5^, as done, for example, for the human endometrial transcriptome^17^. On the other hand, ML algorithms have emerged as useful tools to recognize patterns in data generated by “omics” assays^4^. Here, we demonstrated the power of combining data integration and ML methods to detect endometrial genes whose expression patterns potentially identify a receptive endometrium at around seven days of the estrous cycle.

In the cow, the pre-implantation period is so critical that more than 70% of pregnancy failure associated with embryo death occurs here^18^. This represents one of the main causes of economic loss and thus, the understanding of the early physiological changes occurring in the endometrium, which are determined by variations in the endometrial transcriptome, takes major importance. In the present study, we integrated endometrial transcriptomic data from the *Bos taurus taurus* during this early-luteal phase, with the main aim of identifying a group or set of genes characterizing a receptive endometrium despite the breed and category. Data from *Bos taurus indicus* were not considered to avoid confounding differences given the bifurcation in the phylogenetic tree. Selected datasets (Table 1) shared similar experimental designs. Except for the dataset GSE29853, the other datasets classified the animals retrospectively according to pregnancy status after the biopsy (although GSE107741 was based on the results before and after the biopsy). For the dataset GSE29853, the authors classified the animals based on the results of previous AI. Therefore, all these public datasets have in common that consist of endometrial samples obtained at around day 7 of the estrous cycle, samples were classified according to pregnancy status, and the transcriptome was measured through a high-throughput technology. The application of bioinformatics procedures allowed the integration of these datasets, in the sense that technical differences given by the platform employed for transcriptomic measurement, or the experiment itself, were eliminated through a data pre-processing step (Supplemental Fig. 3).

As per our main objectives, we applied a series of ML procedures (that included identification of sets of genes and application of unsupervised and supervised ML tools to determine the best one) to determine a group of 50 genes with the capability to predict pregnancy status according to their expression levels (Table 2). Even more, the expressions of these genes in all the samples but a particular breed were able to predict uterine receptivity with an overall 96.1% accuracy, validating the predictive capability of these key genes (Table 3).

Between them, there were six transcriptional regulators, corresponding to four transcription factors and two co-factors. Furthermore, five of these 50 genes have been associated with cow fertility in recent studies using genome-wide association analysis. YWHAQ and PAXIP1 were identified as master regulators (molecules that have indirect relationships to positional candidate genes through upstream regulators), and SUCLG2 as one positional candidate gene, associated with dairy heifer fertility^19^. TP53, one of the transcription factors discussed below, was one of the three top upstream regulators of positional candidate genes, while MYH10 a regulator target gene, associated with beef cattle fertility^20^.

It is well accepted that progesterone concentrations regulate the endometrial expression of genes determining uterine receptivity, and it plays a key role in pregnancy establishment and conceptus development^21,22^. Therefore, to explore the action of this hormone on the expression of these 50 genes, additional external data from two studies were re-analyzed. In one study, pregnant heifers presented normal or high levels of progesterone from day 3 of the estrous cycle^23^. Although all heifers were pregnant, only one from the high-progesterone group was classified as such according to the expression of the 50 genes (Fig. 1A). Forde et al.^23^ concluded that progesterone supplementation during early pregnancy advances endometrial gene expression in cattle, and so the endometria of those animals probably reflected changes occurring later in the estrous cycle. In the other study, ovariectomized cows were treated with progesterone for six days, receiving an injection of estradiol benzoate at the end of the treatment or not^24^. Only samples obtained from animals receiving the estradiol treatment were classified as receptive, according to the expression of the 50 genes (Fig. 1B).

Thus, these results suggest that the expression of the 50 genes is temporally regulated and their differences in expression between R and nonR animals would occur at around day 7 of the estrous cycle but not later in the cycle. Also, these genes probably are responding to the increasing estradiol levels of the first wave together with the increased levels of progesterone^25^, but not to progesterone alone, although this fact should be confirmed by experimental studies in vivo.

In addition, and to explore more deeply the biological significance of the 50 genes, related genes were inferred in a network analysis (Supplementary Fig. 2). These related genes might not behave as crucial genes during the early luteal phase period, but their expression, or their products, or the pathway(s) they shared with the key genes, could be affected later. We cannot know exactly the number of genes that would be regulated by the key genes, and so the arbitrary top 100 genes were explored, together with the significantly enriched biological processes by these genes, respectively for the biomarker genes that showed higher or lower expression in the R cows.

One of the biomarker genes with higher expression in the R cows was TP53, which is well known as tumor suppressor because its protein (p53) regulates cell division by keeping cells from growing and dividing (proliferating) too fast or in an uncontrolled way. In response to different stress signals, p53 can hold cell division in both the G1/S phase and G2/M phase checkpoints, in order to prevent chromosomal replication specifically during the cell cycle if DNA damage is present, and even to induce cell apoptosis^26,27^. The actions of p53 are critical to avoid tumor development, but it also regulates many cellular processes, including metabolism, antioxidant response, and DNA repair^26^. Interestingly, regulation of transcription and cell death/apoptosis were biological processes enriched by the related genes to the ones with higher expression in the R cows, while M phase of the cell cycle, chromosome segregation and lipid oxidation to the ones with lower expression in the R cows. Furthermore, many steps involved in implantation in the human, such as apoptosis and angiogenesis, are regulated by p53 and thus this protein could play a broader role in the survival of the specie by optimizing the embryo implantation^28^. This study suggests that early expression by TP53 is critical for uterine receptivity in the cow as well. In the report from Ponsuksili et al.^9^, the authors found that activated TP53 was associated with the endometria of high receptive cows, although on day 3 (not on day 7). Thus, the role of TP53 expression during the early-luteal phase in the bovine endometrium deserves further investigation.

Another biological process enriched with the related genes to the ones with higher expression in the R cows, was Wnt signaling pathway. The key genes involved in it were TLE4 and ROR2. On the other hand, CTBP2, a transcription co-factor with higher expression in the nonR cows, also participates in the Wnt pathway. TLE4 is a transcriptional co-repressor whose products, and the ones encoded by the TLE1-3, inhibit the transcriptional activation mediated by the nuclear β-catenin CTNNB1 and TCF family members in the canonical Wnt signaling pathway. Conversely, HHEX, a transcription factor more expressed in the R cows, acts early in embryo development to enhance canonical WNT-signaling by repressing expression of TLE4^29^. ROR2 signals through a Wnt responsive, β-catenin independent pathway and suppress a canonical Wnt/β-catenin signal^30^. Finally, CTBP2 associates with major components of the β-catenin destruction complex and limits the accessibility of β-catenin to core transcription factors in undifferentiated embryonic stem cells, which allows exit from pluripotency^31^. In the cow, maternally derived Wnt are important for the development of the preimplantation embryo^32^. Therefore, the expression of these biomarker genes identified in the present study could play a crucial role in the regulation of the canonical and non-canonical Wnt pathway in the early-diestrous endometrium.

Lastly, one more biological process that deserves attention is the circadian rhythm, influenced by the transcription factor BHLHE40. The basic helix-loop-helix protein encoded by this gene interacts with the clock genes and modulates the circadian phase of the clock genes, playing a role in the fine regulation and robustness of the molecular clock^33,34^. This clock is highly important in reproductive tissues, including the regulation of the uterine function, although more studies are needed to define its role in the endometrial receptivity (reviewed by Sen and Hoffmann^35^).

*Study strengths and limitations*: This work embraces the output from five studies employing four breeds with distinct purposes (dairy, beef and double) and different techniques for sample collection, which are factors that can influence endometrial gene expression. However, the relative difference of expression between R and nonR animals, for all the biomarker genes (except for five) was similar in all the breeds *before* correction for the experimental effect (Supplementary Fig. 4). In other words, if samples are taken from a given breed cattle, using the same technique, and at around day 7 of the estrous cycle, these genes are expected to show differences in expression between R and nonR animals. Even when the differences were subtle, the overall behavior of these key genes would help to define those animals with a higher uterine capacity to support pregnancy.

On the other hand, the establishment of pregnancy is a complex process that depends not only on the receptive endometrium but also on embryonic viability and synchrony of actions between both parts (reviewed by Spenser et al.^21^). Therefore, we cannot expect that the sole expression of these 50 genes, identified by mathematical approaches, could determine animals that would become pregnant or not which such high accuracy. However, we believe that our results could be of enormous help to understand the characteristics of a receptive endometrium at the time of ET and provide the basis for further studies.

## Conclusion

In summary, the application of supervised and unsupervised ML approaches for multi-transcriptomic data integration and target/gene selection, allowed the identification of a group of 50 endometrial genes with high predictive capability (96.1%) to define uterine receptivity in Taurine cattle at around seven days of the estrous cycle, despite the animal’s breed and category. From a data science perspective, results show the scope and power of ML methods in multi-transcriptomic studies and from a biological perspective, results highlight the concept of the strong influence of the maternal environment for pregnancy establishment, which is determined independently of the presence of the embryo.

## Methodology

### High-throughput datasets

Five transcriptomic datasets were downloaded from a public functional genomic data repository: Gene Expression Omnibus (GEO) from the National Center for Biotechnology Information^36,37^. These studies were selected because they all have in common that endometrial samples from *Bos taurus taurus* animals were obtained at day 6–7 of the estrous cycle, and they were classified as pregnant (n = 26) or not (n = 26) after ET or AI, depending on the study. The accession number and main characteristics of each dataset are shown in Table 1.

Only our dataset (GSE115756,^7^) used the RNA-sequencing technology (Illumina HiSeq 2500 platform). The other datasets measured gene expression through the microarray technology. The study GSE107741 used the Agilent-023647 B. taurus (Bovine) Oligo Microarray v2 while the other three (GSE29853, GSE36080, GSE20974) employed the Affymetrix Bovine Genome Array platform.

### Data integration

The R software platform^38^ was employed in the following procedures. The raw counts obtained from the RNA-sequencing in our data were transformed through the variance stabilizing transformation method^39^, using the vst function from the DESeq2 package for R^40^. This transformation removes the dependence of the variance on the mean and produces transformed data on the log2 scale, which has been normalized with respect to library size or other normalization factors. The raw data obtained from samples hybridized to the Affymetrix or Agilent platforms were processed with the gcRMA^41^ or limma^42^ packages, respectively. Data were imported into R, background corrected, and then transformed and normalized using the quantile normalization method. Next, rows of each data set were collapsed, in order to retain the microarray probe with the highest mean value from the group of the genes with the same Ensembl ID.

Therefore, a table with transformed and normalized gene expression values for each sample was generated for each of the five studies, using the same identifier for the transcripts (Ensembl ID). These tables were integrated into a single table containing the expression of 9850 annotated transcripts for the 52 samples in total (only transcripts with expression values for all the samples were retained). Next, the batch effects (i.e., the fact that the data were obtained from different studies) were removed with the ComBat function from the sva package^43^. A multidimensional scaling analysis (MDS) was employed to evaluate between samples similarities before and after the batch removal (Supplemental Fig. 3), with the Glimma package^44^.

### Selection of biomarker genes through supervised ML

The details about each step followed by the BioDiscML software^12^ are specified in the reference and in the GitHub page (https://github.com/mickaelleclercq/BioDiscML). Briefly, a first sampling step separates the data into a train and a test set (2/3 and 1/3, respectively, by default), that are later used to assess the model, or the user can define these datasets. We chose this last option instead of using a random separation of the data in order to have samples from all the breeds on each set. The training set consisted of 34 samples (11 from Holstein, 7 from Japanese Black, and 8 from Charolais x Limousine, or Simmental cows, respectively). The test set consisted of 18 samples (6 from Holstein, 4 from Japanese Black, and 4 from Charolais x Limousine, or Simmental cows, respectively).

As second step, a feature-ranking algorithm sorts the features (or genes) based on their predictive power with respect to the class (R or nonR), retaining only the best 1000 genes. Next, two methods are employed for searching and selecting the potential biomarker genes: top *k* features and stepwise, for each ML algorithm and each optimization evaluation criterion. At each iteration, the created model is evaluated by tenfold cross validation and the selected genes are retained if the predictive performance is improved. When the signatures of biomarker genes are identified, the models are evaluated again. Finally, it is possible to let the software to select the best model (or combine the best ones), or this step can be done manually. For this, one of the output files describes each model with its associated performance metrics and the list of corresponding genes.

For this study, we manually selected those models that resulted with a prediction accuracy higher than 90% in the test set and the following evaluations’ procedures in the train set: tenfold cross validation, leave-one-out cross validation, repeated holdout and bootstrapping; and repeated holdout in the whole set.

### Identification of the best group of biomarker genes through unsupervised ML

The groups of genes identified as potential biomarkers were evaluated for their ability to blindly cluster apart the R and nonR samples according to their expression levels. For this, a hierarchical clustering was employed, using Spearman Rank Correlation as similarity metric and complete linkage as clustering method, implemented with the Cluster 3.0 software^45^. The resulting dendrogram and the heat map were visualized with Java TreeView^46^.

The correct clustering of the R and nonR samples for each group of genes was evaluated using a confusion matrix, selecting the genes that, according to their expression, presented the highest accuracy to cluster apart the samples from each group.

### Validation of the selected set of biomarker genes through supervised ML

Once the set of potential biomarker genes was selected according to unsupervised learning, the next step was to verify if the expression levels of these genes were able to predict pregnancy status. For this, we applied a different ML model than the ones identified by the BioDiscML software, using Support vector machines (Support vector classifier) with linear kernels (SVM). This method was chosen because of its ability to learn well with only a very small number of features, its robustness against the error of models, and its computational efficiency compared to other ML methods ^47^. In addition, SVM has been shown to successfully classify cancer tissue samples based on gene expression, from microarray technology^48^ or microarray-RNAseq integrated data^49^.

In order to discern if the expression of these genes would be able to predict pregnancy across all the bovine breeds, the training set consisted of all the samples but the samples from a given breed, which were part of the test set. Therefore, four pairs of training-test sets were used for classification (Simmental heifers from both studies were considered together). In other words, the training sets consisted of all the samples but the ones from Holstein (n = 35), or Japanese Black (n = 41), or Charolais x Limousine (n = 40), or Simmental animals (n = 40). Then, the corresponding test set to each training set were all the samples from the Holstein (n = 17), or Japanese Black (n = 11), or Charolais x Limousine (n = 12), or Simmental animals (n = 12).

The leave-one-out cross validation method was employed as the internal control for the training dataset. The implementation of the SVM with linear kernel was done with the kernlab package^50^, through the caret package^51^ for the R software^38^.

### Exploring the biological significance of the selected biomarker genes

As a final step, we investigated the biological meaning of the 50 genes through two methods: predictions in external datasets and functional/network analysis.

*Predictions in external datasets*: Two datasets were selected to generate the test sets based on the endometrial expression of the 50 biomarker genes. These were GSE33030^23^ and GSE16880^24^. Both studies employed the Affymetrix Bovine Genome Array (GPL2112) as platform. Only samples belonging to pregnant heifers treated or not with a progesterone device from day 3 (n = 5 per group), and those obtained from ovariectomized cows treated with progesterone for 6 days receiving or not an estradiol injection (n = 3 per group), downloaded from GSE33030 or GSE16880, respectively, were analyzed. The raw data were processed with the gcRMA package^41^. Data were imported into R, background corrected, and then transformed and normalized using the quantile normalization method. Next, rows of each data set were collapsed, to retain the microarray probe with the highest mean value from the group of the genes with the same Ensembl ID. The 50 genes were isolated from each dataset to be used as test sets, performing an addon batch effect adjustment of this data with the training data with the bapred package^52^. The training data consisted of the batch-corrected expression of the 50 genes for all the 52 samples described in Table 1. SVM with linear kernels was used as classifier, employing the leave-one-out cross validation method as the internal control, applied with the kernlab package^50^, through the caret package^51^ for the R software^38^.

*Functional/network analysis:* A functional classification of the protein class for each gene was overview with the Panther database^13^. Next, in order to expand the knowledge about the genes related to the biomarker genes, a network analysis with Cytoscape V. 3.7.2^14^ was performed.

For this, the Ensembl IDs were converted first to the corresponding human Entrez ID homologous using bioDBnet (https://biodbnet-abcc.ncifcrf.gov/db/db2db.php). Then, the GeneMania plugin^53^, which infers network data, was employed to generate two networks: one for the group of genes increasing in expression, and other for the genes decreasing in expression, in the R cows. The set of functional association data between genes was downloaded from the Homo sapiens database. The up-regulated -or down-regulated- biomarker genes were imported into the GeneMania plugin to retrieve the corresponding association network, allowing the program to find the top 100 related genes. The association data employed was genetic or physical interaction (i.e., two genes are functionally associated, if the effects of perturbing one gene were found to be modified by perturbations to a second gene, or if their products were found to interact in a protein–protein interaction study) or if the genes were in the same reaction within a pathway. Finally, the BinGO plugin^54^ was applied to find the statistically overrepresented biological processes in the resulting networks.

## Supplementary information

Supplementary Information.

## Data Availability

All data are fully resourced from public NCBI GEO databases.
