# Supplementary material for: Machine learning approach to integrated endometrial transcriptomic datasets reveals biomarkers predicting uterine receptivity in cattle at seven days after estrous
Source: Sci Rep. 2020 Oct 12;10:16981. doi: 10.1038/s41598-020-72988-3 (PMC7550564; doi:10.1038/s41598-020-72988-3)

**Supplementary Materials**

**Machine learning approach to integrated endometrial transcriptomic datasets reveals biomarkers predicting uterine receptivity in cattle at seven days after estrous**

Maria B Rabaglino^1^ and Haja N Kadarmideen^1^

^1^Quantitative Genetics, Bioinformatics and Computational Biology Group, Department of Applied Mathematics and Computer Science, Technical University of Denmark, Kemitorvet, 2800 Kgs. Lyngby, Denmark.

***Supplementary Tables 1 and 2, and Figures 1, 2, 3 and 4***

**Supplementary Table 1**. Top 100 genes related with the biomarker genes (A) up-regulated or (B) down-regulated in the animals that became pregnant.

(A) (B)

| **Gene Symbol** | **Gene Name** |  | **Gene Symbol** | **Gene Name** |
| --- | --- | --- | --- | --- |
| AIFM2 | Apoptosis-inducing factor 2 |  | AASS | Alpha-aminoadipic semialdehyde synthase, mitochondrial |
| ALOX12 | Arachidonate 12-lipoxygenase, 12S-type |  | ACADS | Short-chain specific acyl-CoA dehydrogenase, mitochondrial |
| AR | Androgen receptor |  | AK1 | Adenylate kinase isoenzyme 1 |
| ARNT | Aryl hydrocarbon receptor nuclear translocator |  | AMBRA1 | Activating molecule in BECN1-regulated autophagy protein 1 |
| ARNTL | Aryl hydrocarbon receptor nuclear translocator-like protein 1 |  | ARF5 | Auxin response factor 5 |
| ATM | Serine-protein kinase ATM |  | ATG14 | Beclin 1-associated autophagy-related key regulator |
| AUP1 | Ancient ubiquitous protein 1 |  | BECN1 | Beclin-1 |
| BAD | Bcl2-associated agonist of cell death |  | BRCA1 | Breast cancer type 1 susceptibility protein homolog |
| BHLHE41 | Class E basic helix-loop-helix protein 41 |  | CAB39 | Calcium-binding protein 39 |
| BRAF | Serine/threonine-protein kinase B-rafUPF0606 protein KIAA1549 |  | CD5 | T-cell surface glycoprotein CD5 |
| BTG1 | Protein BTG1 |  | CDCA5 | Sororin |
| BTG2 | Protein BTG2 |  | CTBP1 | C-terminal-binding protein 1 |
| CABIN1 | Calcineurin-binding protein cabin-1 |  | CTNNA3 | Catenin alpha-3 |
| CALM1 | Calmodulin 1 |  | CTU1 | Cytoplasmic tRNA 2-thiolation protein 1 |
| CAMK4 | Calcium/calmodulin-dependent protein kinase type IV |  | CWC22 | Pre-mRNA-splicing factor CWC22 |
| CHAF1B | Chromatin assembly factor 1 subunit B |  | CYR61 | Cysteine-rich angiogenic inducer 61 |
| CLOCK | Circadian locomoter output cycles protein kaput |  | DAPP1 | Dual adapter for phosphotyrosine and 3-phosphotyrosine and 3-phosphoinositide |
| CPLX1 | Complexin-1 |  | DDX19B | ATP-dependent RNA helicase DDX19B |
| CRK | Adapter molecule crk |  | EDEM1 | ER degradation-enhancing alpha-mannosidase-like protein 1 |
| CTHRC1 | Collagen triple helix repeat-containing protein 1 |  | EGFLAM | EGF Like, Fibronectin Type III And Laminin G Domains |
| CUL9 | Cullin-9 |  | ETFA | Electron transfer flavoprotein subunit alpha, mitochondrial |
| CUX1 | Homeobox protein cut-like |  | FAM162A | Protein FAM162A |
| DAZAP2 | DAZ-associated protein 2 |  | FAM206A | Protein FAM206A |
| DEC1 | Deleted in esophageal cancer 1 |  | FGFR1 | Fibroblast growth factor receptor |
| DET1 | DET1 homolog |  | FMR1 | Synaptic functional regulator FMR1 |
| DPH2 | 2-(3-amino-3-carboxypropyl)histidine synthase subunit 2 |  | FOXA2 | Hepatocyte nuclear factor 3-beta |
| EGFR | Epidermal growth factor receptorReceptor protein-tyrosine kinase |  | FXR1 | Fragile X mental retardation syndrome-related protein 1 |
| EHD1 | EH domain-containing protein 1 |  | GHITM | Growth hormone-inducible transmembrane protein |
| EIF4A3 | Eukaryotic initiation factor 4A-III |  | GOLPH3 | Golgi phosphoprotein 3 |
| EMID1 | EMI domain-containing protein 1 |  | GOLPH3L | Golgi phosphoprotein 3-like |
| ETV1 | ETS translocation variant 1 |  | GPR143 | G-protein coupled receptor 143 |
| ETV5 | ETS translocation variant 5 |  | HADH | Hydroxyacyl-coenzyme A dehydrogenase, mitochondrial |
| FAS | Tumor necrosis factor receptor superfamily member 6 |  | HDAC8 | Histone deacetylase 8 |
| FOS | Proto-oncogene c-Fos |  | HSD17B10 | 3-hydroxyacyl-CoA dehydrogenase type-2 |
| FZD2 | Frizzled-2 |  | IL17RD | Interleukin-17 receptor D |
| FZD5 | Frizzled-5 |  | IVD | Acyl-CoA_dh_N domain-containing protein |
| HDAC8 | Histone deacetylase 8 |  | KIDINS220 | Kinase D-interacting substrate of 220 kDa |
| HM13 | Minor histocompatibility antigen H13 |  | KLF3 | Krueppel-like factor 3 |
| HSPA1A | Heat shock 70 kDa protein 1A |  | MYH1 | Myosin-1 |
| JMJD6 | Bifunctional arginine demethylase and lysyl-hydroxylase JMJD6 |  | NGF | NGF domain-containing protein |
| KCNK1 | Potassium channel subfamily K member 1 |  | NRIP2 | Nuclear receptor-interacting protein 2 |
| KIF26B | Kinesin-like protein KIF26B |  | PDE4A | cAMP-specific 3',5'-cyclic phosphodiesterase 4A |
| LLGL1 | Lethal(2) giant larvae protein homolog 1 |  | PDS5A | Sister chromatid cohesion protein PDS5 homolog A |
| MAGOH | Protein mago nashi homolog |  | PDS5B | Sister chromatid cohesion protein PDS5 homolog B |
| MAP3K3 | Mitogen-activated protein kinase kinase kinase 3 |  | PGRMC1 | Membrane-associated progesterone receptor component 1 |
| MDM2 | E3 ubiquitin-protein ligase Mdm2 |  | PHLPP2 | PH domain leucine-rich repeat-containing protein phosphatase 2 |
| MDM4 | Protein Mdm4 |  | PIK3CA | Phosphatidylinositol 4,5-bisphosphate 3-kinase catalytic subunit alpha isoform |
| MITF | Microphthalmia-associated transcription factor |  | PIK3R1 | Phosphatidylinositol 3-kinase regulatory subunit alpha |
| MST1R | Macrophage-stimulating protein receptor |  | PIK3R4 | Phosphoinositide 3-kinase regulatory subunit 4 |
| MTOR | Serine/threonine-protein kinase mTOR |  | PLCG1 | 1-phosphatidylinositol 4,5-bisphosphate phosphodiesterase gamma-1 |
| MYB | Transcriptional activator Myb |  | PPARA | Peroxisome proliferator-activated receptor alpha |
| MYO5B | Unconventional myosin-Vb |  | PPM1G | Protein phosphatase 1G |
| NOP14 | Nucleolar protein 14 |  | PPP2R5B | Serine/threonine-protein phosphatase 2A 56 kDa regulatory subunit beta isoform |
| NPAS2 | Neuronal PAS domain-containing protein 2 |  | PRKCI | Protein kinase C iota type |
| NRIP1 | Nuclear receptor-interacting protein 1 |  | PTPN11 | Tyrosine-protein phosphatase non-receptor type 11 |
| NUPR1 | Nuclear protein 1 |  | PTPRF | Receptor-type tyrosine-protein phosphatase F |
| NUSAP1 | Nucleolar and spindle-associated protein 1 |  | QKI | KH domain-containing protein |
| OS9 | Protein OS-9 |  | RAD21 | Double-strand-break repair protein rad21 homolog |
| PAX2 | Paired box protein Pax-2 |  | RAP1A | Ras-related protein Rap-1A |
| PAX6 | Paired box protein Pax-6 |  | RBPJL | Recombining binding protein suppressor of hairless-like protein |
| PAX8 | Paired box protein Pax-8 |  | RBSN | Rabenosyn-5 |
| PDIA2 | Protein disulfide-isomerase A2 |  | REC8 | Meiotic recombination protein REC8 homolog |
| PDX1 | Pancreas/duodenum homeobox protein 1 |  | RUNX1 | Runt-related transcription factor |
| PERP | p53 apoptosis effector related to PMP-22 |  | RXRA | Retinoic acid receptor RXR-alpha |
| PIAS3 | E3 SUMO-protein ligase PIAS3 |  | RXRB | Retinoic acid receptor RXR-beta |
| PIAS4 | E3 SUMO-protein ligase PIAS4 |  | SLC35A2 | UDP-galactose translocator |
| POU2F2 | POU domain, class 2, transcription factor 2 |  | SMC1A | Structural maintenance of chromosomes protein 1A |
| RAB3A | Ras-related protein Rab-3A |  | SMC1B | Structural maintenance of chromosomes protein 1B |
| RANBP2 | E3 SUMO-protein ligase RanBP2 |  | SMC3 | Structural maintenance of chromosomes protein 3 |
| RANGAP1 | Ran GTPase-activating protein 1 |  | SNX1 | Sorting nexin-1 |
| RBM15 | RNA-binding protein 15 |  | SOD1 | Superoxide dismutase [Cu-Zn] |
| RPA2 | Replication protein A 32 kDa subunit |  | SOX13 | Transcription factor SOX-13 |
| SESN1 | Sestrin-1 |  | SOX6 | Transcription factor SOX-6 |
| SH3BP4 | SH3 domain-binding protein 4 |  | SPRY2 | Protein sprouty homolog 2 |
| SIN3A | Paired amphipathic helix protein Sin3a |  | SRRM1 | Serine/arginine repetitive matrix protein 1 |
| SIX3 | Homeobox protein SIX3 |  | STAG1 | STAG domain-containing protein |
| SIX6 | Homeobox protein SIX6 |  | STRAP | Serine-threonine kinase receptor-associated protein |
| SKA1 | Spindle and kinetochore-associated protein 1 |  | SUCLG1 | Succinate--CoA ligase [ADP/GDP-forming] subunit alpha, mitochondrial |
| SLC17A7 | Vesicular glutamate transporter 1 |  | SUFU | Suppressor of fused homolog |
| SLC18A2 | Synaptic vesicular amine transporter |  | TBC1D5 | TBC1 domain family member 5 |
| SLC18A3 | Vesicular acetylcholine transporter |  | TBKBP1 | TANK-binding kinase 1-binding protein 1 |
| SLC32A1 | Vesicular inhibitory amino acid transporter |  | TLE2 | Transducin-like enhancer protein 2 |
| STEAP3 | Metalloreductase STEAP3 |  | TLE3 | Transducin-like enhancer protein 3 |
| STK40 | Serine/threonine-protein kinase 40 |  | TLE4 | Transducin-like enhancer protein 4 |
| SUMO1 | Small ubiquitin-related modifier 1 |  | TLR9 | Toll-like receptor 9 |
| SV2A | Synaptic vesicle glycoprotein 2A |  | TOB1 | Protein Tob1 |
| SYT4 | Synaptotagmin-4 |  | TP53 | Cellular tumor antigen p53 |
| TCEB3 | Transcription elongation factor B polypeptide 3 |  | TPTE | Putative tyrosine-protein phosphatase TPTE |
| TCF7L2 | Transcription factor 7-like 2 |  | UBE2E2 | Ubiquitin-conjugating enzyme E2 E2 |
| TFAP2A | Transcription factor AP-2-alpha |  | UBR7 | Putative E3 ubiquitin-protein ligase UBR7 |
| TMED4 | Transmembrane emp24 domain-containing protein 4 |  | ULK2 | Serine/threonine-protein kinase ULK2 |
| TP53BP1 | TP53-binding protein 1 |  | USP43 | Ubiquitin carboxyl-terminal hydrolase 43 |
| TP53I3 | Quinone oxidoreductase PIG3 |  | VPS29 | Vacuolar protein sorting-associated protein 29 |
| TP63 | Tumor protein p63 |  | VPS35 | Vacuolar protein sorting-associated protein 35 |
| TP73 | Tumor protein p73 |  | WAPL | Wings apart-like protein homolog |
| TRIB3 | Tribbles homolog 3 |  | WDFY1 | WD repeat and FYVE domain-containing protein 1 |
| UBE2J1 | Ubiquitin-conjugating enzyme E2 J1 |  | WDR48 | WD repeat-containing protein 48 |
| VAMP2 | Vesicle-associated membrane protein 2 |  | ZBP1 | Z-DNA-binding protein 1 |
| WNT5A | Protein Wnt-5a |  | ZEB1 | Zinc finger E-box-binding homeobox 1 |
| WWOX | WW domain-containing oxidoreductase |  | ZKSCAN3 | Zinc finger protein with KRAB and SCAN domains 3 |

**Supplementary Table 2.** Biological processes enriched with biomarker genes (A) increasing or (B) decreasing in expression in the cows that became pregnant, and their related genes.

(A)

| **GO-ID** | **p-value** | **corr p-value** | **Description** | **Genes in test set** |
| --- | --- | --- | --- | --- |
| 65007 | 8.28E-14 | 1.25E-10 | biological regulation | SMARCAL1\|RAB3A\|EIF4A3\|SESN1\|MAGOH\|MYB\|NUSAP1\|TP63\|PIAS4\|TLE4\|PDIA2\|PIAS3\|WNT5A\|MITF\|JMJD6\|AR\|SCG5\|TRIB3\|RIN3\|NUPR1\|ROR2\|TRIB1\|CLOCK\|TP53\|PPIC\|CUL9\|ALOX12\|MST1R\|SKA1\|NPAS2\|UBE2J1\|SKA2\|COP1\|TSPAN6\|TP53BP1\|MYH10\|TFAP2A\|SYT4\|FZD2\|UBE2I\|FZD5\|SYT1\|BRAF\|POU2F2\|EHD1\|TP53I3\|MYO5B\|BHLHE40\|MDM2\|MDM4\|ATM\|CALM1\|TP73\|BTG2\|BTG1\|BHLHE41\|DEC1\|SIX6\|CHAF1B\|HHEX\|CHAF1A\|SUMO1\|YWHAQ\|SIN3A\|AIFM2\|SIX3\|TMED4\|RBM15\|ARNT\|PAX6\|ETV1\|FOS\|RANGAP1\|PAX2\|ETV5\|PAX8\|CRK\|HDAC8\|EGFR\|ARNTL\|OS9\|CUX1\|SV2A\|PERP\|NRIP1\|TCEB3\|SLC17A7\|MAP3K3\|WWOX\|TCF7L2\|COL26A1\|BAD\|PDX1\|MTOR\|CAMK4\|FAS\|HSPA1A |
| 48518 | 1.47E-13 | 1.25E-10 | positive regulation of biological process | BTG2\|BTG1\|EIF4A3\|HHEX\|SUMO1\|AIFM2\|SIX3\|NUSAP1\|TP63\|TMED4\|PIAS4\|PIAS3\|RBM15\|WNT5A\|ARNT\|MITF\|FOS\|PAX2\|AR\|PAX8\|TRIB3\|NUPR1\|ROR2\|TRIB1\|CLOCK\|TP53\|ALOX12\|MST1R\|NPAS2\|EGFR\|ARNTL\|TSPAN6\|PERP\|NRIP1\|TP53BP1\|TFAP2A\|MAP3K3\|WWOX\|TCF7L2\|COL26A1\|BAD\|PDX1\|BRAF\|MTOR\|EHD1\|TP53I3\|MDM2\|FAS\|MDM4\|ATM\|CALM1\|TP73 |
| 50789 | 4.49E-13 | 2.55E-10 | regulation of biological process | SMARCAL1\|BTG2\|RAB3A\|BTG1\|EIF4A3\|BHLHE41\|DEC1\|SIX6\|CHAF1B\|HHEX\|CHAF1A\|SUMO1\|YWHAQ\|SIN3A\|AIFM2\|SESN1\|MAGOH\|MYB\|SIX3\|NUSAP1\|TP63\|TMED4\|PIAS4\|TLE4\|PDIA2\|PIAS3\|RBM15\|WNT5A\|ARNT\|MITF\|PAX6\|ETV1\|FOS\|RANGAP1\|PAX2\|ETV5\|JMJD6\|AR\|PAX8\|SCG5\|TRIB3\|RIN3\|NUPR1\|ROR2\|TRIB1\|CLOCK\|TP53\|CRK\|PPIC\|CUL9\|ALOX12\|MST1R\|HDAC8\|SKA1\|NPAS2\|EGFR\|UBE2J1\|SKA2\|ARNTL\|COP1\|CUX1\|TSPAN6\|PERP\|NRIP1\|TCEB3\|TP53BP1\|MYH10\|TFAP2A\|MAP3K3\|WWOX\|TCF7L2\|COL26A1\|FZD2\|UBE2I\|FZD5\|SYT1\|BAD\|PDX1\|BRAF\|POU2F2\|MTOR\|EHD1\|TP53I3\|MYO5B\|CAMK4\|BHLHE40\|MDM2\|FAS\|MDM4\|ATM\|CALM1\|TP73\|HSPA1A |
| 50794 | 8.56E-13 | 3.65E-10 | regulation of cellular process | SMARCAL1\|BTG2\|RAB3A\|BTG1\|EIF4A3\|BHLHE41\|DEC1\|SIX6\|CHAF1B\|HHEX\|CHAF1A\|SUMO1\|YWHAQ\|SIN3A\|AIFM2\|SESN1\|MAGOH\|MYB\|SIX3\|NUSAP1\|TP63\|TMED4\|PIAS4\|TLE4\|PDIA2\|PIAS3\|RBM15\|WNT5A\|ARNT\|MITF\|PAX6\|ETV1\|FOS\|RANGAP1\|PAX2\|ETV5\|JMJD6\|AR\|PAX8\|SCG5\|TRIB3\|RIN3\|NUPR1\|ROR2\|TRIB1\|CLOCK\|TP53\|CRK\|PPIC\|CUL9\|ALOX12\|MST1R\|HDAC8\|SKA1\|NPAS2\|EGFR\|SKA2\|ARNTL\|COP1\|CUX1\|TSPAN6\|PERP\|NRIP1\|TCEB3\|TP53BP1\|MYH10\|TFAP2A\|MAP3K3\|WWOX\|TCF7L2\|COL26A1\|FZD2\|UBE2I\|FZD5\|SYT1\|BAD\|PDX1\|BRAF\|POU2F2\|MTOR\|TP53I3\|CAMK4\|BHLHE40\|MDM2\|FAS\|MDM4\|ATM\|CALM1\|TP73\|HSPA1A |
| 48522 | 1.18E-12 | 4.03E-10 | positive regulation of cellular process | BTG1\|EIF4A3\|ALOX12\|MST1R\|NPAS2\|EGFR\|ARNTL\|HHEX\|SUMO1\|AIFM2\|TSPAN6\|PERP\|SIX3\|NRIP1\|NUSAP1\|TP53BP1\|TP63\|TMED4\|PIAS4\|PIAS3\|MAP3K3\|WWOX\|TCF7L2\|COL26A1\|RBM15\|BAD\|WNT5A\|PDX1\|ARNT\|MITF\|BRAF\|FOS\|PAX2\|MTOR\|AR\|TP53I3\|PAX8\|MDM2\|FAS\|TRIB3\|MDM4\|ATM\|NUPR1\|ROR2\|TRIB1\|CLOCK\|TP53\|TP73 |
| 6357 | 6.51E-11 | 1.60E-08 | regulation of transcription from RNA polymerase II promoter | SMARCAL1\|HDAC8\|NPAS2\|ARNTL\|HHEX\|CUX1\|SIN3A\|SIX3\|NRIP1\|TCEB3\|TP63\|PIAS4\|TFAP2A\|TCF7L2\|RBM15\|PDX1\|ARNT\|MITF\|FOS\|AR\|PAX8\|MDM2\|MDM4\|ROR2\|CLOCK\|TP53\|CRK |
| 10468 | 6.55E-11 | 1.60E-08 | regulation of gene expression | SMARCAL1\|BTG2\|BTG1\|EIF4A3\|BHLHE41\|DEC1\|SIX6\|CHAF1B\|HHEX\|CHAF1A\|SUMO1\|YWHAQ\|SIN3A\|MAGOH\|MYB\|SIX3\|TP63\|PIAS4\|TLE4\|PIAS3\|RBM15\|WNT5A\|ARNT\|MITF\|PAX6\|ETV1\|FOS\|PAX2\|ETV5\|JMJD6\|AR\|PAX8\|TRIB3\|ROR2\|TRIB1\|CLOCK\|TP53\|CRK\|ALOX12\|HDAC8\|NPAS2\|ARNTL\|CUX1\|NRIP1\|TCEB3\|TP53BP1\|TFAP2A\|TCF7L2\|UBE2I\|PDX1\|POU2F2\|MTOR\|BHLHE40\|MDM2\|MDM4\|TP73 |
| 10604 | 9.75E-11 | 2.08E-08 | positive regulation of macromolecule metabolic process | EIF4A3\|ALOX12\|NPAS2\|ARNTL\|HHEX\|SUMO1\|SIX3\|NRIP1\|TP53BP1\|TP63\|PIAS4\|PIAS3\|TCF7L2\|RBM15\|WNT5A\|PDX1\|ARNT\|MITF\|FOS\|PAX2\|MTOR\|AR\|PAX8\|MDM2\|TRIB3\|ROR2\|TRIB1\|CLOCK\|TP53\|TP73 |
| 10556 | 1.12E-10 | 2.13E-08 | regulation of macromolecule biosynthetic process | SMARCAL1\|BTG2\|BTG1\|EIF4A3\|BHLHE41\|DEC1\|SIX6\|CHAF1B\|HHEX\|CHAF1A\|SUMO1\|YWHAQ\|SIN3A\|MAGOH\|MYB\|SIX3\|TP63\|PIAS4\|TLE4\|PIAS3\|RBM15\|WNT5A\|ARNT\|MITF\|PAX6\|ETV1\|FOS\|PAX2\|ETV5\|JMJD6\|AR\|PAX8\|TRIB3\|ROR2\|TRIB1\|CLOCK\|TP53\|CRK\|HDAC8\|NPAS2\|ARNTL\|CUX1\|NRIP1\|TCEB3\|TP53BP1\|TFAP2A\|TCF7L2\|UBE2I\|PDX1\|POU2F2\|MTOR\|BHLHE40\|MDM2\|MDM4\|TP73 |
| 45449 | 1.36E-10 | 2.15E-08 | regulation of transcription | SMARCAL1\|BTG2\|BTG1\|BHLHE41\|DEC1\|SIX6\|CHAF1B\|HHEX\|CHAF1A\|SUMO1\|YWHAQ\|SIN3A\|MYB\|SIX3\|TP63\|PIAS4\|TLE4\|PIAS3\|RBM15\|WNT5A\|ARNT\|MITF\|PAX6\|ETV1\|FOS\|PAX2\|ETV5\|JMJD6\|AR\|PAX8\|TRIB3\|ROR2\|TRIB1\|CLOCK\|TP53\|CRK\|HDAC8\|NPAS2\|ARNTL\|CUX1\|NRIP1\|TCEB3\|TP53BP1\|TFAP2A\|TCF7L2\|UBE2I\|PDX1\|POU2F2\|BHLHE40\|MDM2\|MDM4\|TP73 |
| 9893 | 1.39E-10 | 2.15E-08 | positive regulation of metabolic process | EIF4A3\|ALOX12\|NPAS2\|EGFR\|ARNTL\|HHEX\|SUMO1\|SIX3\|NRIP1\|TP53BP1\|TP63\|PIAS4\|PIAS3\|TCF7L2\|RBM15\|WNT5A\|PDX1\|ARNT\|MITF\|FOS\|PAX2\|MTOR\|AR\|PAX8\|MDM2\|TRIB3\|ROR2\|TRIB1\|CLOCK\|TP53\|TP73 |
| 31325 | 1.85E-10 | 2.63E-08 | positive regulation of cellular metabolic process | EIF4A3\|NPAS2\|EGFR\|ARNTL\|HHEX\|SUMO1\|SIX3\|NRIP1\|TP53BP1\|TP63\|PIAS4\|PIAS3\|TCF7L2\|RBM15\|WNT5A\|PDX1\|ARNT\|MITF\|FOS\|PAX2\|MTOR\|AR\|PAX8\|MDM2\|TRIB3\|ROR2\|TRIB1\|CLOCK\|TP53\|TP73 |
| 31326 | 2.26E-10 | 2.96E-08 | regulation of cellular biosynthetic process | SMARCAL1\|BTG2\|BTG1\|EIF4A3\|BHLHE41\|DEC1\|SIX6\|CHAF1B\|HHEX\|CHAF1A\|SUMO1\|YWHAQ\|SIN3A\|MAGOH\|MYB\|SIX3\|TP63\|PIAS4\|TLE4\|PIAS3\|RBM15\|WNT5A\|ARNT\|MITF\|PAX6\|ETV1\|FOS\|PAX2\|ETV5\|JMJD6\|AR\|PAX8\|TRIB3\|ROR2\|TRIB1\|CLOCK\|TP53\|CRK\|HDAC8\|NPAS2\|EGFR\|ARNTL\|CUX1\|NRIP1\|TCEB3\|TP53BP1\|TFAP2A\|TCF7L2\|UBE2I\|PDX1\|POU2F2\|MTOR\|BHLHE40\|MDM2\|MDM4\|TP73 |
| 9889 | 3.10E-10 | 3.77E-08 | regulation of biosynthetic process | SMARCAL1\|BTG2\|BTG1\|EIF4A3\|BHLHE41\|DEC1\|SIX6\|CHAF1B\|HHEX\|CHAF1A\|SUMO1\|YWHAQ\|SIN3A\|MAGOH\|MYB\|SIX3\|TP63\|PIAS4\|TLE4\|PIAS3\|RBM15\|WNT5A\|ARNT\|MITF\|PAX6\|ETV1\|FOS\|PAX2\|ETV5\|JMJD6\|AR\|PAX8\|TRIB3\|ROR2\|TRIB1\|CLOCK\|TP53\|CRK\|HDAC8\|NPAS2\|EGFR\|ARNTL\|CUX1\|NRIP1\|TCEB3\|TP53BP1\|TFAP2A\|TCF7L2\|UBE2I\|PDX1\|POU2F2\|MTOR\|BHLHE40\|MDM2\|MDM4\|TP73 |
| 51252 | 1.45E-09 | 1.65E-07 | regulation of RNA metabolic process | SMARCAL1\|BHLHE41\|HDAC8\|NPAS2\|ARNTL\|DEC1\|SIX6\|HHEX\|YWHAQ\|CUX1\|SIN3A\|MYB\|SIX3\|NRIP1\|TCEB3\|TP53BP1\|TP63\|PIAS4\|TFAP2A\|TCF7L2\|UBE2I\|RBM15\|PDX1\|ARNT\|MITF\|PAX6\|ETV1\|FOS\|POU2F2\|ETV5\|JMJD6\|AR\|PAX8\|BHLHE40\|MDM2\|MDM4\|ROR2\|CLOCK\|TP53\|CRK\|TP73 |
| 60255 | 1.86E-09 | 1.98E-07 | regulation of macromolecule metabolic process | SMARCAL1\|BTG2\|BTG1\|EIF4A3\|BHLHE41\|DEC1\|SIX6\|CHAF1B\|HHEX\|CHAF1A\|SUMO1\|YWHAQ\|SIN3A\|MAGOH\|MYB\|SIX3\|TP63\|PIAS4\|TLE4\|PIAS3\|RBM15\|WNT5A\|ARNT\|MITF\|PAX6\|ETV1\|FOS\|PAX2\|ETV5\|JMJD6\|AR\|PAX8\|TRIB3\|ROR2\|TRIB1\|CLOCK\|TP53\|CRK\|ALOX12\|HDAC8\|NPAS2\|EGFR\|UBE2J1\|ARNTL\|CUX1\|NRIP1\|TCEB3\|TP53BP1\|TFAP2A\|TCF7L2\|UBE2I\|PDX1\|POU2F2\|MTOR\|BHLHE40\|MDM2\|MDM4\|TP73 |
| 6355 | 2.38E-09 | 2.39E-07 | regulation of transcription, DNA-dependent | SMARCAL1\|BHLHE41\|HDAC8\|NPAS2\|ARNTL\|DEC1\|SIX6\|HHEX\|YWHAQ\|CUX1\|SIN3A\|MYB\|SIX3\|NRIP1\|TCEB3\|TP53BP1\|TP63\|PIAS4\|TFAP2A\|TCF7L2\|UBE2I\|RBM15\|PDX1\|ARNT\|MITF\|PAX6\|ETV1\|FOS\|POU2F2\|ETV5\|AR\|PAX8\|BHLHE40\|MDM2\|MDM4\|ROR2\|CLOCK\|TP53\|CRK\|TP73 |
| 51171 | 3.15E-09 | 2.98E-07 | regulation of nitrogen compound metabolic process | SMARCAL1\|BTG2\|BTG1\|BHLHE41\|DEC1\|SIX6\|CHAF1B\|HHEX\|CHAF1A\|SUMO1\|YWHAQ\|SIN3A\|MYB\|SIX3\|TP63\|PIAS4\|TLE4\|PIAS3\|RBM15\|WNT5A\|ARNT\|MITF\|PAX6\|ETV1\|FOS\|PAX2\|ETV5\|JMJD6\|AR\|PAX8\|TRIB3\|ROR2\|TRIB1\|CLOCK\|TP53\|CRK\|HDAC8\|NPAS2\|EGFR\|ARNTL\|CUX1\|NRIP1\|TCEB3\|TP53BP1\|TFAP2A\|TCF7L2\|UBE2I\|PDX1\|POU2F2\|MTOR\|BHLHE40\|MDM2\|MDM4\|TP73 |
| 10628 | 4.27E-09 | 3.73E-07 | positive regulation of gene expression | TCF7L2\|RBM15\|WNT5A\|PDX1\|ARNT\|MITF\|ALOX12\|FOS\|NPAS2\|PAX2\|ARNTL\|AR\|HHEX\|PAX8\|SIX3\|NRIP1\|ROR2\|TP53BP1\|CLOCK\|TP53\|TP63\|TP73 |
| 31328 | 4.38E-09 | 3.73E-07 | positive regulation of cellular biosynthetic process | TCF7L2\|RBM15\|EIF4A3\|WNT5A\|PDX1\|ARNT\|MITF\|FOS\|NPAS2\|PAX2\|EGFR\|MTOR\|ARNTL\|AR\|HHEX\|PAX8\|SIX3\|NRIP1\|ROR2\|TP53BP1\|CLOCK\|TP53\|TP63\|TP73 |
| 45892 | 4.83E-09 | 3.92E-07 | negative regulation of transcription, DNA-dependent | PIAS4\|TCF7L2\|UBE2I\|RBM15\|PDX1\|HDAC8\|DEC1\|HHEX\|YWHAQ\|CUX1\|SIN3A\|BHLHE40\|SIX3\|NRIP1\|MDM2\|MDM4\|TP53\|TP63 |
| 9891 | 5.89E-09 | 4.55E-07 | positive regulation of biosynthetic process | TCF7L2\|RBM15\|EIF4A3\|WNT5A\|PDX1\|ARNT\|MITF\|FOS\|NPAS2\|PAX2\|EGFR\|MTOR\|ARNTL\|AR\|HHEX\|PAX8\|SIX3\|NRIP1\|ROR2\|TP53BP1\|CLOCK\|TP53\|TP63\|TP73 |
| 51253 | 6.35E-09 | 4.55E-07 | negative regulation of RNA metabolic process | PIAS4\|TCF7L2\|UBE2I\|RBM15\|PDX1\|HDAC8\|DEC1\|HHEX\|YWHAQ\|CUX1\|SIN3A\|BHLHE40\|SIX3\|NRIP1\|MDM2\|MDM4\|TP53\|TP63 |
| 10557 | 6.41E-09 | 4.55E-07 | positive regulation of macromolecule biosynthetic process | TCF7L2\|RBM15\|EIF4A3\|WNT5A\|PDX1\|ARNT\|MITF\|FOS\|NPAS2\|PAX2\|MTOR\|ARNTL\|AR\|HHEX\|PAX8\|SIX3\|NRIP1\|ROR2\|TP53BP1\|CLOCK\|TP53\|TP63\|TP73 |
| 19219 | 7.18E-09 | 4.90E-07 | regulation of nucleobase, nucleoside, nucleotide and nucleic acid metabolic process | SMARCAL1\|BTG2\|BTG1\|BHLHE41\|DEC1\|SIX6\|CHAF1B\|HHEX\|CHAF1A\|SUMO1\|YWHAQ\|SIN3A\|MYB\|SIX3\|TP63\|PIAS4\|TLE4\|PIAS3\|RBM15\|WNT5A\|ARNT\|MITF\|PAX6\|ETV1\|FOS\|PAX2\|ETV5\|JMJD6\|AR\|PAX8\|TRIB3\|ROR2\|TRIB1\|CLOCK\|TP53\|CRK\|HDAC8\|NPAS2\|ARNTL\|CUX1\|NRIP1\|TCEB3\|TP53BP1\|TFAP2A\|TCF7L2\|UBE2I\|PDX1\|POU2F2\|MTOR\|BHLHE40\|MDM2\|MDM4\|TP73 |
| 45941 | 9.93E-09 | 6.51E-07 | positive regulation of transcription | TCF7L2\|RBM15\|WNT5A\|PDX1\|ARNT\|MITF\|FOS\|NPAS2\|PAX2\|ARNTL\|AR\|HHEX\|PAX8\|SIX3\|NRIP1\|ROR2\|TP53BP1\|CLOCK\|TP53\|TP63\|TP73 |
| 48523 | 1.47E-08 | 9.30E-07 | negative regulation of cellular process | BTG2\|BTG1\|EIF4A3\|ALOX12\|HDAC8\|EGFR\|DEC1\|HHEX\|SUMO1\|YWHAQ\|CUX1\|SIN3A\|SESN1\|SIX3\|NRIP1\|TP63\|PIAS4\|WWOX\|TCF7L2\|UBE2I\|RBM15\|WNT5A\|PDX1\|ARNT\|MITF\|BRAF\|MTOR\|AR\|BHLHE40\|MDM2\|FAS\|TRIB3\|MDM4\|ATM\|ROR2\|TRIB1\|TP53\|TP73\|HSPA1A |
| 45944 | 2.15E-08 | 1.31E-06 | positive regulation of transcription from RNA polymerase II promoter | TCF7L2\|RBM15\|PDX1\|ARNT\|MITF\|FOS\|NPAS2\|ARNTL\|AR\|HHEX\|PAX8\|SIX3\|NRIP1\|ROR2\|CLOCK\|TP53\|TP63 |
| 16481 | 2.49E-08 | 1.46E-06 | negative regulation of transcription | PIAS4\|TCF7L2\|UBE2I\|RBM15\|PDX1\|HDAC8\|DEC1\|HHEX\|SUMO1\|YWHAQ\|CUX1\|SIN3A\|BHLHE40\|SIX3\|NRIP1\|MDM2\|MDM4\|TP53\|TP63 |
| 6836 | 2.77E-08 | 1.56E-06 | neurotransmitter transport | SYT4\|RAB3A\|SYT1\|SLC32A1\|SV2A\|SLC17A7\|CPLX1\|SLC18A2\|SLC18A3 |
| 45893 | 2.92E-08 | 1.56E-06 | positive regulation of transcription, DNA-dependent | TCF7L2\|RBM15\|PDX1\|ARNT\|MITF\|FOS\|NPAS2\|ARNTL\|AR\|HHEX\|PAX8\|SIX3\|NRIP1\|ROR2\|TP53BP1\|CLOCK\|TP53\|TP63\|TP73 |
| 31327 | 2.93E-08 | 1.56E-06 | negative regulation of cellular biosynthetic process | PIAS4\|TCF7L2\|UBE2I\|RBM15\|EIF4A3\|PDX1\|HDAC8\|DEC1\|HHEX\|SUMO1\|YWHAQ\|CUX1\|SIN3A\|BHLHE40\|SIX3\|NRIP1\|MDM2\|TRIB3\|MDM4\|TP53\|TP63 |
| 51173 | 3.37E-08 | 1.74E-06 | positive regulation of nitrogen compound metabolic process | TCF7L2\|RBM15\|WNT5A\|PDX1\|ARNT\|MITF\|FOS\|NPAS2\|PAX2\|EGFR\|ARNTL\|AR\|HHEX\|PAX8\|SIX3\|NRIP1\|ROR2\|TP53BP1\|CLOCK\|TP53\|TP63\|TP73 |
| 51254 | 3.53E-08 | 1.77E-06 | positive regulation of RNA metabolic process | TCF7L2\|RBM15\|PDX1\|ARNT\|MITF\|FOS\|NPAS2\|ARNTL\|AR\|HHEX\|PAX8\|SIX3\|NRIP1\|ROR2\|TP53BP1\|CLOCK\|TP53\|TP63\|TP73 |
| 9890 | 3.87E-08 | 1.88E-06 | negative regulation of biosynthetic process | PIAS4\|TCF7L2\|UBE2I\|RBM15\|EIF4A3\|PDX1\|HDAC8\|DEC1\|HHEX\|SUMO1\|YWHAQ\|CUX1\|SIN3A\|BHLHE40\|SIX3\|NRIP1\|MDM2\|TRIB3\|MDM4\|TP53\|TP63 |
| 80090 | 4.08E-08 | 1.93E-06 | regulation of primary metabolic process | SMARCAL1\|BTG2\|BTG1\|EIF4A3\|BHLHE41\|DEC1\|SIX6\|CHAF1B\|HHEX\|CHAF1A\|SUMO1\|YWHAQ\|SIN3A\|MAGOH\|MYB\|SIX3\|TP63\|PIAS4\|TLE4\|PIAS3\|RBM15\|WNT5A\|ARNT\|MITF\|PAX6\|ETV1\|FOS\|PAX2\|ETV5\|JMJD6\|AR\|PAX8\|TRIB3\|ROR2\|TRIB1\|CLOCK\|TP53\|CRK\|HDAC8\|NPAS2\|EGFR\|UBE2J1\|ARNTL\|CUX1\|NRIP1\|TCEB3\|TP53BP1\|TFAP2A\|TCF7L2\|UBE2I\|PDX1\|POU2F2\|MTOR\|BHLHE40\|MDM2\|MDM4\|TP73 |
| 48519 | 5.70E-08 | 2.63E-06 | negative regulation of biological process | BTG2\|BTG1\|EIF4A3\|ALOX12\|HDAC8\|EGFR\|DEC1\|HHEX\|SUMO1\|YWHAQ\|CUX1\|SIN3A\|SESN1\|SIX3\|NRIP1\|TP63\|PIAS4\|WWOX\|TCF7L2\|UBE2I\|RBM15\|WNT5A\|PDX1\|ARNT\|MITF\|BRAF\|MTOR\|AR\|BHLHE40\|MDM2\|FAS\|TRIB3\|MDM4\|ATM\|ROR2\|TRIB1\|CALM1\|TP53\|TP73\|HSPA1A |
| 10558 | 8.91E-08 | 3.97E-06 | negative regulation of macromolecule biosynthetic process | PIAS4\|TCF7L2\|UBE2I\|RBM15\|EIF4A3\|PDX1\|HDAC8\|DEC1\|HHEX\|SUMO1\|YWHAQ\|CUX1\|SIN3A\|BHLHE40\|SIX3\|NRIP1\|MDM2\|MDM4\|TP53\|TP63 |
| 51641 | 9.30E-08 | 3.97E-06 | cellular localization | STEAP3\|RAB3A\|CPLX1\|EGFR\|ARNTL\|HHEX\|YWHAQ\|OS9\|CUX1\|SIN3A\|SIX3\|NUSAP1\|MYH10\|RANBP2\|PDIA2\|SYT4\|SYT1\|WNT5A\|PDX1\|ARNT\|EHD1\|SCG5\|MDM2\|TP53\|LLGL1\|VAMP2 |
| 45935 | 9.32E-08 | 3.97E-06 | positive regulation of nucleobase, nucleoside, nucleotide and nucleic acid metabolic process | TCF7L2\|RBM15\|WNT5A\|PDX1\|ARNT\|MITF\|FOS\|NPAS2\|PAX2\|ARNTL\|AR\|HHEX\|PAX8\|SIX3\|NRIP1\|ROR2\|TP53BP1\|CLOCK\|TP53\|TP63\|TP73 |
| 10629 | 1.39E-07 | 5.76E-06 | negative regulation of gene expression | PIAS4\|TCF7L2\|UBE2I\|RBM15\|PDX1\|HDAC8\|DEC1\|HHEX\|SUMO1\|YWHAQ\|CUX1\|SIN3A\|BHLHE40\|SIX3\|NRIP1\|MDM2\|MDM4\|TP53\|TP63 |
| 45934 | 1.59E-07 | 6.47E-06 | negative regulation of nucleobase, nucleoside, nucleotide and nucleic acid metabolic process | PIAS4\|TCF7L2\|UBE2I\|RBM15\|PDX1\|HDAC8\|DEC1\|HHEX\|SUMO1\|YWHAQ\|CUX1\|SIN3A\|BHLHE40\|SIX3\|NRIP1\|MDM2\|MDM4\|TP53\|TP63 |
| 51172 | 1.88E-07 | 7.46E-06 | negative regulation of nitrogen compound metabolic process | PIAS4\|TCF7L2\|UBE2I\|RBM15\|PDX1\|HDAC8\|DEC1\|HHEX\|SUMO1\|YWHAQ\|CUX1\|SIN3A\|BHLHE40\|SIX3\|NRIP1\|MDM2\|MDM4\|TP53\|TP63 |
| 7623 | 3.38E-07 | 1.31E-05 | circadian rhythm | DEC1\|BHLHE40\|BHLHE41\|CLOCK\|NPAS2\|EGFR\|ARNTL |
| 31324 | 4.73E-07 | 1.79E-05 | negative regulation of cellular metabolic process | PIAS4\|TCF7L2\|UBE2I\|RBM15\|EIF4A3\|PDX1\|HDAC8\|MTOR\|DEC1\|HHEX\|SUMO1\|YWHAQ\|CUX1\|SIN3A\|BHLHE40\|SIX3\|NRIP1\|MDM2\|TRIB3\|MDM4\|TP53\|TP63 |
| 19222 | 5.47E-07 | 2.02E-05 | regulation of metabolic process | SMARCAL1\|BTG2\|BTG1\|EIF4A3\|BHLHE41\|DEC1\|SIX6\|CHAF1B\|HHEX\|CHAF1A\|SUMO1\|YWHAQ\|SIN3A\|MAGOH\|MYB\|SIX3\|TP63\|PIAS4\|TLE4\|PIAS3\|RBM15\|WNT5A\|ARNT\|MITF\|PAX6\|ETV1\|FOS\|PAX2\|ETV5\|JMJD6\|AR\|PAX8\|TRIB3\|ROR2\|TRIB1\|CLOCK\|TP53\|CRK\|ALOX12\|HDAC8\|NPAS2\|EGFR\|UBE2J1\|ARNTL\|CUX1\|NRIP1\|TCEB3\|TP53BP1\|TFAP2A\|TCF7L2\|UBE2I\|PDX1\|POU2F2\|MTOR\|BHLHE40\|MDM2\|MDM4\|TP73 |
| 31323 | 6.72E-07 | 2.43E-05 | regulation of cellular metabolic process | SMARCAL1\|BTG2\|BTG1\|EIF4A3\|BHLHE41\|DEC1\|SIX6\|CHAF1B\|HHEX\|CHAF1A\|SUMO1\|YWHAQ\|SIN3A\|MAGOH\|MYB\|SIX3\|TP63\|PIAS4\|TLE4\|PIAS3\|RBM15\|WNT5A\|ARNT\|MITF\|PAX6\|ETV1\|FOS\|PAX2\|ETV5\|JMJD6\|AR\|PAX8\|TRIB3\|ROR2\|TRIB1\|CLOCK\|TP53\|CRK\|HDAC8\|NPAS2\|EGFR\|ARNTL\|CUX1\|NRIP1\|TCEB3\|TP53BP1\|TFAP2A\|TCF7L2\|UBE2I\|PDX1\|POU2F2\|MTOR\|BHLHE40\|MDM2\|MDM4\|TP73 |
| 122 | 7.35E-07 | 2.61E-05 | negative regulation of transcription from RNA polymerase II promoter | PIAS4\|TCF7L2\|RBM15\|PDX1\|HDAC8\|HHEX\|CUX1\|SIN3A\|NRIP1\|MDM2\|MDM4\|TP53\|TP63 |
| 42981 | 1.78E-06 | 6.21E-05 | regulation of apoptosis | WWOX\|TCF7L2\|BTG2\|BTG1\|BAD\|WNT5A\|MITF\|ALOX12\|BRAF\|EGFR\|COP1\|TP53I3\|SIN3A\|AIFM2\|PERP\|FAS\|ATM\|NUPR1\|TP53\|TP63\|TP73\|HSPA1A |
| 43067 | 2.08E-06 | 7.09E-05 | regulation of programmed cell death | WWOX\|TCF7L2\|BTG2\|BTG1\|BAD\|WNT5A\|MITF\|ALOX12\|BRAF\|EGFR\|COP1\|TP53I3\|SIN3A\|AIFM2\|PERP\|FAS\|ATM\|NUPR1\|TP53\|TP63\|TP73\|HSPA1A |
| 9892 | 2.29E-06 | 7.65E-05 | negative regulation of metabolic process | PIAS4\|TCF7L2\|UBE2I\|RBM15\|EIF4A3\|PDX1\|HDAC8\|MTOR\|DEC1\|HHEX\|SUMO1\|YWHAQ\|CUX1\|SIN3A\|BHLHE40\|SIX3\|NRIP1\|MDM2\|TRIB3\|MDM4\|TP53\|TP63 |
| 10941 | 2.38E-06 | 7.79E-05 | regulation of cell death | WWOX\|TCF7L2\|BTG2\|BTG1\|BAD\|WNT5A\|MITF\|ALOX12\|BRAF\|EGFR\|COP1\|TP53I3\|SIN3A\|AIFM2\|PERP\|FAS\|ATM\|NUPR1\|TP53\|TP63\|TP73\|HSPA1A |
| 43066 | 2.87E-06 | 9.24E-05 | negative regulation of apoptosis | TCF7L2\|BTG2\|WNT5A\|MITF\|ALOX12\|BRAF\|EGFR\|SIN3A\|FAS\|ATM\|TP53\|TP63\|TP73\|HSPA1A |
| 60571 | 3.08E-06 | 9.71E-05 | morphogenesis of an epithelial fold | AR\|HHEX\|TP63\|EGFR |
| 43069 | 3.35E-06 | 1.04E-04 | negative regulation of programmed cell death | TCF7L2\|BTG2\|WNT5A\|MITF\|ALOX12\|BRAF\|EGFR\|SIN3A\|FAS\|ATM\|TP53\|TP63\|TP73\|HSPA1A |
| 33554 | 3.46E-06 | 1.05E-04 | cellular response to stress | BTG2\|WNT5A\|ARNT\|RPA2\|FOS\|ETV5\|CHAF1B\|CHAF1A\|SUMO1\|OS9\|SESN1\|ATM\|ROR2\|TP53BP1\|TRIB1\|TP53\|TP63\|TP73 |
| 60548 | 4.26E-06 | 1.27E-04 | negative regulation of cell death | TCF7L2\|BTG2\|WNT5A\|MITF\|ALOX12\|BRAF\|EGFR\|SIN3A\|FAS\|ATM\|TP53\|TP63\|TP73\|HSPA1A |
| 51246 | 4.93E-06 | 1.45E-04 | regulation of protein metabolic process | PIAS4\|PIAS3\|UBE2I\|EIF4A3\|WNT5A\|ARNT\|EGFR\|MTOR\|UBE2J1\|ARNTL\|SUMO1\|MAGOH\|MDM2\|TRIB3\|MDM4\|TRIB1\|TP53\|TP73 |
| 2347 | 5.55E-06 | 1.58E-04 | response to tumor cell | TP53\|TP63\|TP73 |
| 60601 | 5.55E-06 | 1.58E-04 | lateral sprouting from an epithelium | AR\|WNT5A\|TP63 |
| 42127 | 6.10E-06 | 1.70E-04 | regulation of cell proliferation | BTG2\|BTG1\|WNT5A\|PDX1\|ARNT\|MITF\|ALOX12\|MST1R\|EGFR\|MTOR\|DEC1\|AR\|HHEX\|SESN1\|MDM2\|MDM4\|ROR2\|TRIB1\|TP53\|TP63\|HSPA1A |
| 10646 | 7.55E-06 | 2.07E-04 | regulation of cell communication | RAB3A\|EGFR\|DEC1\|HHEX\|TSPAN6\|SIX3\|TP63\|TMED4\|MAP3K3\|WWOX\|TCF7L2\|WNT5A\|ARNT\|BRAF\|MTOR\|AR\|BHLHE40\|SCG5\|MDM2\|TRIB3\|ATM\|TRIB1\|TP53\|CRK\|TP73 |
| 10605 | 9.64E-06 | 2.61E-04 | negative regulation of macromolecule metabolic process | PIAS4\|TCF7L2\|UBE2I\|RBM15\|EIF4A3\|PDX1\|HDAC8\|DEC1\|HHEX\|SUMO1\|YWHAQ\|CUX1\|SIN3A\|BHLHE40\|SIX3\|NRIP1\|MDM2\|MDM4\|TP53\|TP63 |
| 33233 | 1.10E-05 | 2.89E-04 | regulation of protein sumoylation | PIAS4\|PIAS3\|ARNT |
| 33235 | 1.10E-05 | 2.89E-04 | positive regulation of protein sumoylation | PIAS4\|PIAS3\|ARNT |
| 16055 | 1.27E-05 | 3.27E-04 | Wnt receptor signaling pathway | PIAS4\|TLE4\|TCF7L2\|FZD2\|FZD5\|WNT5A\|MITF\|ROR2 |
| 35468 | 1.65E-05 | 4.19E-04 | positive regulation of signaling pathway | MAP3K3\|TCF7L2\|WNT5A\|ARNT\|BRAF\|EGFR\|MTOR\|AR\|HHEX\|TSPAN6\|ATM\|TP63\|TMED4 |
| 43193 | 1.86E-05 | 4.66E-04 | positive regulation of gene-specific transcription | AR\|TCF7L2\|HHEX\|RBM15\|ARNT\|TP53\|TP63\|TP73 |
| 9649 | 1.92E-05 | 4.74E-04 | entrainment of circadian clock | DEC1\|BHLHE40\|CLOCK |
| 9887 | 1.95E-05 | 4.74E-04 | organ morphogenesis | TFAP2A\|WWOX\|TCF7L2\|RBM15\|WNT5A\|BHLHE41\|PDX1\|BRAF\|PAX6\|PAX2\|EGFR\|AR\|SIX6\|HHEX\|SIX3\|ROR2\|TP63 |
| 51649 | 2.83E-05 | 6.80E-04 | establishment of localization in cell | RANBP2\|SYT4\|STEAP3\|RAB3A\|SYT1\|PDX1\|ARNT\|CPLX1\|ARNTL\|EHD1\|HHEX\|YWHAQ\|CUX1\|SIX3\|NUSAP1\|SCG5\|TP53\|MYH10\|LLGL1\|VAMP2 |
| 35112 | 3.05E-05 | 7.13E-04 | genitalia morphogenesis | TCF7L2\|ROR2\|TP63 |
| 7164 | 3.05E-05 | 7.13E-04 | establishment of tissue polarity | FZD2\|FZD5\|TP63 |
| 42176 | 3.42E-05 | 7.87E-04 | regulation of protein catabolic process | SUMO1\|WNT5A\|MDM2\|MDM4\|TRIB1\|ARNTL |
| 51179 | 3.69E-05 | 8.39E-04 | localization | STEAP3\|RAB3A\|BTG1\|EIF4A3\|CPLX1\|EGFR\|ARNTL\|HHEX\|YWHAQ\|OS9\|CUX1\|SIN3A\|SV2A\|MAGOH\|SIX3\|NRIP1\|NUSAP1\|SH3BP4\|SLC17A7\|MYH10\|SLC18A2\|TMED4\|SLC18A3\|CTHRC1\|RANBP2\|PDIA2\|SYT4\|SYT1\|SLC32A1\|WNT5A\|PDX1\|ARNT\|JMJD6\|EHD1\|AR\|MYO5B\|SCG5\|MDM2\|RIN3\|KCNK1\|TP53\|LLGL1\|VAMP2\|TP73 |
| 33365 | 3.75E-05 | 8.40E-04 | protein localization in organelle | RANBP2\|PDIA2\|HHEX\|OS9\|SIX3\|MDM2\|TP53\|ARNTL |
| 10647 | 3.93E-05 | 8.69E-04 | positive regulation of cell communication | MAP3K3\|TCF7L2\|WNT5A\|ARNT\|BRAF\|EGFR\|MTOR\|AR\|HHEX\|TSPAN6\|ATM\|TP63\|TMED4 |
| 48511 | 4.12E-05 | 8.99E-04 | rhythmic process | DEC1\|BHLHE40\|NRIP1\|BHLHE41\|CLOCK\|NPAS2\|EGFR\|ARNTL |
| 51247 | 5.03E-05 | 1.08E-03 | positive regulation of protein metabolic process | PIAS4\|PIAS3\|SUMO1\|EIF4A3\|WNT5A\|MDM2\|ARNT\|TRIB3\|TRIB1\|TP53\|MTOR |
| 65008 | 5.12E-05 | 1.09E-03 | regulation of biological quality | RAB3A\|BTG1\|ALOX12\|DEC1\|OS9\|SV2A\|SLC17A7\|MYH10\|TP63\|PDIA2\|SYT4\|TCF7L2\|FZD2\|SYT1\|BAD\|PDX1\|MTOR\|JMJD6\|EHD1\|AR\|BHLHE40\|SCG5\|MDM2\|MDM4\|NUPR1\|TP53\|TP73\|HSPA1A |
| 9653 | 5.45E-05 | 1.15E-03 | anatomical structure morphogenesis | TFAP2A\|WWOX\|TCF7L2\|RAB3A\|FZD2\|RBM15\|FZD5\|WNT5A\|BHLHE41\|PDX1\|BRAF\|PAX6\|PAX2\|EGFR\|AR\|SIX6\|HHEX\|PAX8\|SIX3\|ATM\|ROR2\|TP53\|TP63\|TP73 |
| 2009 | 5.53E-05 | 1.15E-03 | morphogenesis of an epithelium | TFAP2A\|AR\|HHEX\|RBM15\|WNT5A\|PDX1\|TP63\|PAX2\|EGFR |
| 48546 | 5.92E-05 | 1.22E-03 | digestive tract morphogenesis | TCF7L2\|WNT5A\|TP63\|TP73 |
| 6917 | 6.34E-05 | 1.28E-03 | induction of apoptosis | WWOX\|TP53I3\|AIFM2\|BAD\|PERP\|FAS\|ATM\|NUPR1\|TP53\|TP63\|TP73 |
| 34613 | 6.37E-05 | 1.28E-03 | cellular protein localization | RANBP2\|PDIA2\|WNT5A\|EGFR\|ARNTL\|HHEX\|YWHAQ\|OS9\|SIN3A\|SIX3\|SCG5\|MDM2\|TP53 |
| 12502 | 6.52E-05 | 1.29E-03 | induction of programmed cell death | WWOX\|TP53I3\|AIFM2\|BAD\|PERP\|FAS\|ATM\|NUPR1\|TP53\|TP63\|TP73 |
| 70727 | 6.83E-05 | 1.30E-03 | cellular macromolecule localization | RANBP2\|PDIA2\|WNT5A\|EGFR\|ARNTL\|HHEX\|YWHAQ\|OS9\|SIN3A\|SIX3\|SCG5\|MDM2\|TP53 |
| 30538 | 6.87E-05 | 1.30E-03 | embryonic genitalia morphogenesis | TCF7L2\|ROR2 |
| 6335 | 6.87E-05 | 1.30E-03 | DNA replication-dependent nucleosome assembly | CHAF1B\|CHAF1A |
| 60599 | 6.87E-05 | 1.30E-03 | lateral sprouting involved in mammary gland duct morphogenesis | AR\|WNT5A |
| 8629 | 6.98E-05 | 1.30E-03 | induction of apoptosis by intracellular signals | TP53I3\|ATM\|TP53\|TP63\|TP73 |
| 16265 | 7.00E-05 | 1.30E-03 | death | PDIA2\|DIDO1\|WWOX\|STEAP3\|BAD\|JMJD6\|AR\|AIFM2\|PERP\|FAS\|TRIB3\|MDM4\|ATM\|TP53\|TP63\|SLC18A2\|TP73 |
| 35466 | 7.43E-05 | 1.36E-03 | regulation of signaling pathway | MAP3K3\|WWOX\|TCF7L2\|WNT5A\|ARNT\|BRAF\|EGFR\|MTOR\|AR\|HHEX\|TSPAN6\|SIX3\|MDM2\|TRIB3\|ATM\|TRIB1\|TP53\|CRK\|TP63\|TP73\|TMED4 |
| 8285 | 7.48E-05 | 1.36E-03 | negative regulation of cell proliferation | DEC1\|AR\|BTG2\|BTG1\|SESN1\|WNT5A\|PDX1\|MDM4\|ROR2\|TRIB1\|TP53\|HSPA1A |
| 8361 | 8.86E-05 | 1.59E-03 | regulation of cell size | AR\|BTG1\|ALOX12\|NUPR1\|TP53\|TP63\|MTOR\|TP73\|HSPA1A |
| 48806 | 9.22E-05 | 1.64E-03 | genitalia development | TCF7L2\|WNT5A\|ROR2\|TP63 |
| 7049 | 9.62E-05 | 1.69E-03 | cell cycle | TCF7L2\|STEAP3\|UBE2I\|SKA1\|EGFR\|SKA2\|CHAF1B\|HHEX\|CHAF1A\|SIN3A\|SESN1\|NUSAP1\|MDM2\|MDM4\|ATM\|TP53\|MYH10\|TP73 |
| 10740 | 9.84E-05 | 1.71E-03 | positive regulation of intracellular protein kinase cascade | AR\|MAP3K3\|TCF7L2\|TSPAN6\|WNT5A\|BRAF\|EGFR\|MTOR\|TMED4 |
| 34504 | 1.24E-04 | 2.13E-03 | protein localization in nucleus | RANBP2\|HHEX\|SIX3\|MDM2\|TP53\|ARNTL |
| 32270 | 1.53E-04 | 2.61E-03 | positive regulation of cellular protein metabolic process | PIAS4\|PIAS3\|SUMO1\|EIF4A3\|MDM2\|ARNT\|TRIB3\|TRIB1\|TP53\|MTOR |
| 8630 | 1.55E-04 | 2.61E-03 | DNA damage response, signal transduction resulting in induction of apoptosis | ATM\|TP53\|TP63\|TP73 |
| 55123 | 1.75E-04 | 2.93E-03 | digestive system development | TCF7L2\|WNT5A\|PDX1\|TP63\|TP73 |
| 9987 | 1.94E-04 | 3.21E-03 | cellular process | SMARCAL1\|DIDO1\|STEAP3\|BTG2\|RAB3A\|BTG1\|EIF4A3\|BHLHE41\|CABIN1\|CHAF1B\|HHEX\|CHAF1A\|SMPD4\|SUMO1\|YWHAQ\|SIN3A\|AIFM2\|SESN1\|MAGOH\|SIX3\|NUSAP1\|TP63\|SLC18A2\|SLC18A3\|PDIA2\|WNT5A\|ARNT\|MITF\|PAX6\|ETV1\|FOS\|PAX2\|ETV5\|JMJD6\|AR\|PAX8\|SCG5\|TRIB3\|RIN3\|NUPR1\|ROR2\|TRIB1\|TP53\|CRK\|LLGL1\|VAMP2\|STK40\|PPIC\|CUL9\|ALOX12\|MST1R\|HDAC8\|CPLX1\|SKA1\|EGFR\|UBE2J1\|SKA2\|ARNTL\|OS9\|CUX1\|SV2A\|DPH2\|PERP\|TCEB3\|SH3BP4\|SLC17A7\|TP53BP1\|MYH10\|CTHRC1\|RANBP2\|SYT4\|MAP3K3\|WWOX\|TCF7L2\|NOP14\|FZD2\|UBE2I\|FZD5\|SYT1\|BAD\|PDX1\|RPA2\|BRAF\|POU2F2\|MTOR\|CLK2\|EHD1\|TP53I3\|KIF26B\|CAMK4\|MDM2\|FAS\|MDM4\|ATM\|TP73\|HSPA1A |
| 45603 | 2.05E-04 | 3.33E-03 | positive regulation of endothelial cell differentiation | BTG1\|ALOX12 |
| 34723 | 2.05E-04 | 3.33E-03 | DNA replication-dependent nucleosome organization | CHAF1B\|CHAF1A |
| 8219 | 2.15E-04 | 3.46E-03 | cell death | PDIA2\|DIDO1\|WWOX\|STEAP3\|BAD\|JMJD6\|AR\|AIFM2\|PERP\|FAS\|TRIB3\|MDM4\|ATM\|TP53\|TP63\|TP73 |
| 32268 | 2.24E-04 | 3.57E-03 | regulation of cellular protein metabolic process | PIAS4\|PIAS3\|EIF4A3\|WNT5A\|ARNT\|EGFR\|MTOR\|SUMO1\|MAGOH\|MDM2\|TRIB3\|TRIB1\|TP53\|TP73 |
| 23052 | 2.59E-04 | 4.06E-03 | signaling | RAB3A\|SEL1L\|MST1R\|CPLX1\|EGFR\|CABIN1\|YWHAQ\|SIX3\|NRIP1\|SLC17A7\|TP63\|PIAS4\|TLE4\|SYT4\|MAP3K3\|TCF7L2\|FZD2\|RBM15\|FZD5\|SYT1\|BAD\|WNT5A\|PDX1\|MITF\|BRAF\|FOS\|RANGAP1\|MTOR\|JMJD6\|AR\|CAMK4\|SCG5\|FAS\|RIN3\|ATM\|ROR2\|TRIB1\|CALM1\|CLOCK\|TP53\|CRK\|PPIC\|TP73 |
| 6915 | 2.59E-04 | 4.06E-03 | apoptosis | PDIA2\|DIDO1\|WWOX\|STEAP3\|BAD\|JMJD6\|AIFM2\|PERP\|FAS\|TRIB3\|MDM4\|TP53\|TP63\|TP73 |
| 16331 | 2.70E-04 | 4.18E-03 | morphogenesis of embryonic epithelium | TFAP2A\|HHEX\|WNT5A\|PDX1\|TP63 |
| 9896 | 2.89E-04 | 4.43E-03 | positive regulation of catabolic process | SUMO1\|WNT5A\|MDM2\|ARNT\|TRIB1 |
| 12501 | 3.10E-04 | 4.68E-03 | programmed cell death | PDIA2\|DIDO1\|WWOX\|STEAP3\|BAD\|JMJD6\|AIFM2\|PERP\|FAS\|TRIB3\|MDM4\|TP53\|TP63\|TP73 |
| 45792 | 3.10E-04 | 4.68E-03 | negative regulation of cell size | BTG1\|TP53\|TP63\|MTOR\|TP73\|HSPA1A |
| 43065 | 3.13E-04 | 4.68E-03 | positive regulation of apoptosis | WWOX\|TP53I3\|AIFM2\|BAD\|PERP\|FAS\|ALOX12\|ATM\|NUPR1\|TP53\|TP63\|TP73 |
| 32940 | 3.24E-04 | 4.80E-03 | secretion by cell | SYT4\|STEAP3\|RAB3A\|SYT1\|PDX1\|ARNT\|CPLX1\|LLGL1 |
| 43068 | 3.33E-04 | 4.89E-03 | positive regulation of programmed cell death | WWOX\|TP53I3\|AIFM2\|BAD\|PERP\|FAS\|ALOX12\|ATM\|NUPR1\|TP53\|TP63\|TP73 |
| 22402 | 3.38E-04 | 4.93E-03 | cell cycle process | TCF7L2\|UBE2I\|SKA1\|EGFR\|SKA2\|SIN3A\|SESN1\|NUSAP1\|MDM2\|MDM4\|ATM\|TP53\|MYH10\|TP73 |
| 48645 | 3.51E-04 | 5.07E-03 | organ formation | AR\|HHEX\|TP63 |
| 10942 | 3.61E-04 | 5.16E-03 | positive regulation of cell death | WWOX\|TP53I3\|AIFM2\|BAD\|PERP\|FAS\|ALOX12\|ATM\|NUPR1\|TP53\|TP63\|TP73 |
| 10627 | 3.64E-04 | 5.16E-03 | regulation of intracellular protein kinase cascade | AR\|MAP3K3\|TCF7L2\|TSPAN6\|WNT5A\|BRAF\|EGFR\|MTOR\|TP73\|TMED4 |
| 32583 | 3.93E-04 | 5.53E-03 | regulation of gene-specific transcription | AR\|TCF7L2\|HHEX\|RBM15\|ARNT\|TP53\|TP63\|TP73 |
| 281 | 4.08E-04 | 5.65E-03 | cytokinesis after mitosis | NUSAP1\|MYH10 |
| 60157 | 4.08E-04 | 5.65E-03 | urinary bladder development | WNT5A\|TP63 |
| 44260 | 4.20E-04 | 5.78E-03 | cellular macromolecule metabolic process | CUL9\|SMARCAL1\|DIDO1\|BTG2\|BTG1\|EIF4A3\|MST1R\|HDAC8\|EGFR\|UBE2J1\|CHAF1B\|CHAF1A\|SUMO1\|OS9\|SIN3A\|DPH2\|MAGOH\|TCEB3\|TP53BP1\|RANBP2\|PDIA2\|MAP3K3\|NOP14\|UBE2I\|WNT5A\|ARNT\|RPA2\|BRAF\|ETV1\|FOS\|POU2F2\|PAX2\|MTOR\|JMJD6\|CLK2\|AR\|CAMK4\|MDM2\|TRIB3\|ATM\|ROR2\|TP53\|STK40\|PPIC\|TP73\|HSPA1A |
| 6974 | 4.63E-04 | 6.31E-03 | response to DNA damage stimulus | BTG2\|CHAF1B\|CHAF1A\|SUMO1\|SESN1\|RPA2\|ATM\|TP53BP1\|TP53\|TP63\|TP73 |
| 9790 | 4.67E-04 | 6.31E-03 | embryonic development | TFAP2A\|TCF7L2\|WNT5A\|PDX1\|ARNT\|RPA2\|EGFR\|AR\|HHEX\|SIN3A\|ATM\|ROR2\|TP53\|TP63 |
| 45732 | 4.79E-04 | 6.42E-03 | positive regulation of protein catabolic process | SUMO1\|WNT5A\|MDM2\|TRIB1 |
| 48729 | 4.83E-04 | 6.43E-03 | tissue morphogenesis | TFAP2A\|AR\|HHEX\|RBM15\|WNT5A\|PDX1\|TP63\|PAX2\|EGFR |
| 51716 | 4.94E-04 | 6.50E-03 | cellular response to stimulus | BTG2\|WNT5A\|ARNT\|RPA2\|FOS\|ETV5\|CHAF1B\|CHAF1A\|SUMO1\|OS9\|SESN1\|TRIB3\|ATM\|ROR2\|TP53BP1\|TRIB1\|TP53\|TP63\|TP73 |
| 9967 | 4.96E-04 | 6.50E-03 | positive regulation of signal transduction | AR\|MAP3K3\|TCF7L2\|TSPAN6\|WNT5A\|BRAF\|EGFR\|MTOR\|TMED4 |
| 60429 | 5.07E-04 | 6.60E-03 | epithelium development | TFAP2A\|AR\|HHEX\|FZD2\|RBM15\|WNT5A\|PDX1\|TP63\|PAX2\|EGFR |
| 23056 | 5.65E-04 | 7.29E-03 | positive regulation of signaling process | AR\|MAP3K3\|TCF7L2\|TSPAN6\|WNT5A\|BRAF\|EGFR\|MTOR\|TMED4 |
| 32436 | 5.78E-04 | 7.40E-03 | positive regulation of proteasomal ubiquitin-dependent protein catabolic process | SUMO1\|MDM2\|TRIB1 |
| 32535 | 5.94E-04 | 7.56E-03 | regulation of cellular component size | AR\|BTG1\|ALOX12\|NUPR1\|TP53\|TP63\|MTOR\|TP73\|HSPA1A |
| 7223 | 6.70E-04 | 8.46E-03 | Wnt receptor signaling pathway, calcium modulating pathway | FZD2\|WNT5A\|ROR2 |
| 6402 | 7.24E-04 | 9.07E-03 | mRNA catabolic process | EIF4A3\|MAGOH\|ATM\|HSPA1A |
| 35567 | 7.71E-04 | 9.59E-03 | non-canonical Wnt receptor signaling pathway | FZD2\|WNT5A\|ROR2 |
| 46903 | 7.99E-04 | 9.81E-03 | secretion | SYT4\|STEAP3\|RAB3A\|SYT1\|PDX1\|ARNT\|CPLX1\|LLGL1\|TP73 |
| 33036 | 8.00E-04 | 9.81E-03 | macromolecule localization | RANBP2\|PDIA2\|STEAP3\|RAB3A\|EIF4A3\|WNT5A\|ARNT\|EGFR\|ARNTL\|HHEX\|YWHAQ\|OS9\|SIN3A\|MYO5B\|MAGOH\|SIX3\|NRIP1\|SCG5\|MDM2\|TP53 |
| 23033 | 8.09E-04 | 9.85E-03 | signaling pathway | RAB3A\|SEL1L\|MST1R\|EGFR\|CABIN1\|YWHAQ\|NRIP1\|TP63\|PIAS4\|TLE4\|MAP3K3\|TCF7L2\|FZD2\|RBM15\|FZD5\|BAD\|WNT5A\|PDX1\|MITF\|BRAF\|FOS\|MTOR\|JMJD6\|AR\|SCG5\|ATM\|ROR2\|TRIB1\|CALM1\|TP53\|TP73 |
| 90304 | 8.27E-04 | 9.99E-03 | nucleic acid metabolic process | SMARCAL1\|DIDO1\|NOP14\|BTG2\|EIF4A3\|ARNT\|RPA2\|ETV1\|FOS\|POU2F2\|PAX2\|JMJD6\|AR\|CHAF1B\|CHAF1A\|SUMO1\|SIN3A\|MAGOH\|TCEB3\|ATM\|TP53BP1\|TP53\|TP73\|HSPA1A |
| 10033 | 8.40E-04 | 1.01E-02 | response to organic substance | PIAS3\|TCF7L2\|BTG2\|BTG1\|WNT5A\|ALOX12\|BRAF\|FOS\|EGFR\|MTOR\|AR\|HHEX\|TRIB3\|TRIB1\|KCNK1\|SLC18A2\|HSPA1A |
| 43009 | 8.44E-04 | 1.01E-02 | chordate embryonic development | TFAP2A\|AR\|TCF7L2\|SIN3A\|WNT5A\|ARNT\|ATM\|ROR2\|TP53\|EGFR |
| 51897 | 8.82E-04 | 1.04E-02 | positive regulation of protein kinase B signaling cascade | TCF7L2\|EGFR\|MTOR |
| 6281 | 9.00E-04 | 1.06E-02 | DNA repair | BTG2\|CHAF1B\|CHAF1A\|SUMO1\|RPA2\|ATM\|TP53BP1\|TP53\|TP73 |
| 9792 | 9.19E-04 | 1.07E-02 | embryonic development ending in birth or egg hatching | TFAP2A\|AR\|TCF7L2\|SIN3A\|WNT5A\|ARNT\|ATM\|ROR2\|TP53\|EGFR |
| 33043 | 9.37E-04 | 1.09E-02 | regulation of organelle organization | CUL9\|RAB3A\|NUSAP1\|ATM\|TP53\|SKA1\|MTOR\|SKA2 |
| 43434 | 9.56E-04 | 1.10E-02 | response to peptide hormone stimulus | AR\|BTG2\|HHEX\|BTG1\|TRIB3\|BRAF\|MTOR |
| 51234 | 9.78E-04 | 1.12E-02 | establishment of localization | STEAP3\|RAB3A\|EIF4A3\|CPLX1\|ARNTL\|HHEX\|YWHAQ\|CUX1\|SV2A\|MAGOH\|SIX3\|NUSAP1\|SH3BP4\|SLC17A7\|MYH10\|SLC18A2\|TMED4\|SLC18A3\|RANBP2\|SYT4\|SYT1\|SLC32A1\|PDX1\|ARNT\|JMJD6\|EHD1\|AR\|MYO5B\|SCG5\|MDM2\|RIN3\|KCNK1\|TP53\|LLGL1\|VAMP2\|TP73 |
| 45601 | 1.01E-03 | 1.12E-02 | regulation of endothelial cell differentiation | BTG1\|ALOX12 |
| 60572 | 1.01E-03 | 1.12E-02 | morphogenesis of an epithelial bud | HHEX\|TP63 |
| 60685 | 1.01E-03 | 1.12E-02 | regulation of prostatic bud formation | AR\|WNT5A |
| 7442 | 1.01E-03 | 1.12E-02 | hindgut morphogenesis | TCF7L2\|WNT5A |
| 32879 | 1.02E-03 | 1.13E-02 | regulation of localization | TCF7L2\|RAB3A\|SYT1\|WNT5A\|ARNT\|ALOX12\|EGFR\|EHD1\|SUMO1\|MYO5B\|SCG5\|TRIB3\|ROR2\|TRIB1\|CALM1 |
| 31331 | 1.05E-03 | 1.14E-02 | positive regulation of cellular catabolic process | SUMO1\|MDM2\|ARNT\|TRIB1 |
| 48565 | 1.05E-03 | 1.14E-02 | digestive tract development | TCF7L2\|WNT5A\|TP63\|TP73 |
| 42752 | 1.13E-03 | 1.23E-02 | regulation of circadian rhythm | DEC1\|BHLHE40\|CLOCK |
| 1655 | 1.19E-03 | 1.29E-02 | urogenital system development | AR\|PAX8\|WNT5A\|TP63\|PAX2\|JMJD6 |
| 6950 | 1.21E-03 | 1.29E-02 | response to stress | BTG2\|BTG1\|MST1R\|EGFR\|CHAF1B\|HHEX\|CHAF1A\|SUMO1\|OS9\|SESN1\|TP53BP1\|TP63\|PDIA2\|WNT5A\|ARNT\|RPA2\|PAX6\|FOS\|ETV5\|AR\|TRIB3\|ATM\|ROR2\|TRIB1\|TP53\|TP73\|HSPA1A |
| 6259 | 1.24E-03 | 1.32E-02 | DNA metabolic process | SMARCAL1\|BTG2\|CHAF1B\|CHAF1A\|SUMO1\|SIN3A\|RPA2\|ATM\|TP53BP1\|FOS\|TP53\|TP73 |
| 90066 | 1.32E-03 | 1.40E-02 | regulation of anatomical structure size | AR\|BTG1\|ALOX12\|NUPR1\|TP53\|TP63\|MTOR\|TP73\|HSPA1A |
| 9416 | 1.33E-03 | 1.40E-02 | response to light stimulus | DEC1\|BHLHE40\|FOS\|CLOCK\|TP53\|EGFR |
| 51592 | 1.37E-03 | 1.43E-02 | response to calcium ion | SYT1\|WNT5A\|CALM1\|EGFR |
| 6621 | 1.40E-03 | 1.44E-02 | protein retention in ER lumen | PDIA2\|OS9 |
| 35437 | 1.40E-03 | 1.44E-02 | maintenance of protein localization in endoplasmic reticulum | PDIA2\|OS9 |
| 48513 | 1.42E-03 | 1.44E-02 | organ development | RAB3A\|BHLHE41\|EGFR\|SIX6\|HHEX\|SIX3\|NRIP1\|TP63\|TFAP2A\|WWOX\|TCF7L2\|FZD2\|RBM15\|WNT5A\|PDX1\|ARNT\|MITF\|BRAF\|PAX6\|PAX2\|JMJD6\|AR\|PAX8\|ATM\|ROR2\|TP53\|TP73 |
| 32434 | 1.42E-03 | 1.44E-02 | regulation of proteasomal ubiquitin-dependent protein catabolic process | SUMO1\|MDM2\|TRIB1 |
| 61136 | 1.42E-03 | 1.44E-02 | regulation of proteasomal protein catabolic process | SUMO1\|MDM2\|TRIB1 |
| 9952 | 1.43E-03 | 1.44E-02 | anterior/posterior pattern formation | BTG2\|HHEX\|SIX3\|ATM\|ROR2\|TP53 |
| 61138 | 1.47E-03 | 1.48E-02 | morphogenesis of a branching epithelium | AR\|HHEX\|RBM15\|WNT5A\|TP63 |
| 10552 | 1.54E-03 | 1.53E-02 | positive regulation of gene-specific transcription from RNA polymerase II promoter | AR\|TCF7L2\|HHEX\|RBM15\|TP53 |
| 8104 | 1.58E-03 | 1.57E-02 | protein localization | RANBP2\|PDIA2\|STEAP3\|RAB3A\|WNT5A\|ARNT\|EGFR\|ARNTL\|HHEX\|YWHAQ\|OS9\|SIN3A\|MYO5B\|SIX3\|SCG5\|MDM2\|TP53 |
| 43687 | 1.67E-03 | 1.63E-02 | post-translational protein modification | MAP3K3\|BTG2\|UBE2I\|BTG1\|WNT5A\|BRAF\|MST1R\|HDAC8\|EGFR\|MTOR\|JMJD6\|UBE2J1\|CLK2\|SUMO1\|OS9\|CAMK4\|MDM2\|TRIB3\|ATM\|ROR2\|STK40 |
| 30308 | 1.68E-03 | 1.63E-02 | negative regulation of cell growth | BTG1\|TP53\|TP63\|TP73\|HSPA1A |
| 72001 | 1.68E-03 | 1.63E-02 | renal system development | PAX8\|WNT5A\|TP63\|PAX2\|JMJD6 |
| 8637 | 1.75E-03 | 1.70E-02 | apoptotic mitochondrial changes | AIFM2\|TP53\|TP73 |
| 90 | 1.86E-03 | 1.78E-02 | mitotic anaphase | SKA1\|SKA2 |
| 33205 | 1.86E-03 | 1.78E-02 | cell cycle cytokinesis | NUSAP1\|MYH10 |
| 3002 | 1.92E-03 | 1.82E-02 | regionalization | BTG2\|HHEX\|SIX3\|ATM\|ROR2\|TP53\|TP63 |
| 7389 | 1.92E-03 | 1.82E-02 | pattern specification process | BTG2\|HHEX\|RBM15\|SIX3\|ATM\|ROR2\|TP53\|TP63 |
| 42221 | 2.03E-03 | 1.91E-02 | response to chemical stimulus | PDIA2\|PIAS3\|TCF7L2\|BTG2\|BTG1\|SYT1\|WNT5A\|ARNT\|ALOX12\|BRAF\|FOS\|ETV5\|EGFR\|MTOR\|AR\|HHEX\|TRIB3\|TRIB1\|CALM1\|KCNK1\|TP53\|SLC18A2\|HSPA1A |
| 7050 | 2.07E-03 | 1.94E-02 | cell cycle arrest | TCF7L2\|SESN1\|ATM\|TP53\|TP73 |
| 48568 | 2.13E-03 | 1.98E-02 | embryonic organ development | TFAP2A\|TCF7L2\|HHEX\|WNT5A\|ARNT\|ROR2\|EGFR |
| 45595 | 2.19E-03 | 2.03E-02 | regulation of cell differentiation | AR\|TCF7L2\|BTG1\|RBM15\|BAD\|WNT5A\|ARNT\|MITF\|TRIB3\|ALOX12\|TP53\|TP63 |
| 9314 | 2.24E-03 | 2.07E-02 | response to radiation | DEC1\|BHLHE40\|ATM\|FOS\|CLOCK\|TP53\|EGFR |
| 1938 | 2.34E-03 | 2.12E-02 | positive regulation of endothelial cell proliferation | WNT5A\|ARNT\|MTOR |
| 51896 | 2.34E-03 | 2.12E-02 | regulation of protein kinase B signaling cascade | TCF7L2\|EGFR\|MTOR |
| 50793 | 2.34E-03 | 2.12E-02 | regulation of developmental process | TFAP2A\|TCF7L2\|BTG1\|RBM15\|BAD\|WNT5A\|ARNT\|MITF\|ALOX12\|AR\|HHEX\|TRIB3\|TP53\|MYH10\|TP63 |
| 6350 | 2.36E-03 | 2.12E-02 | transcription | AR\|DIDO1\|TCEB3\|ARNT\|RPA2\|ETV1\|POU2F2\|TP53\|PAX2 |
| 30154 | 2.37E-03 | 2.12E-02 | cell differentiation | BTG2\|RAB3A\|BHLHE41\|EGFR\|HHEX\|SIX3\|TP63\|WWOX\|TCF7L2\|FZD2\|FZD5\|WNT5A\|PDX1\|ARNT\|MITF\|PAX6\|PAX2\|MTOR\|JMJD6\|AR\|PAX8\|ATM\|ROR2\|TP53\|TP73 |
| 71478 | 2.38E-03 | 2.12E-02 | cellular response to radiation | ATM\|TP53 |
| 9894 | 2.41E-03 | 2.14E-02 | regulation of catabolic process | SUMO1\|WNT5A\|MDM2\|ARNT\|MDM4\|TRIB1\|MTOR\|ARNTL |
| 1505 | 2.46E-03 | 2.18E-02 | regulation of neurotransmitter levels | SYT4\|RAB3A\|SYT1\|SLC17A7 |
| 30856 | 2.56E-03 | 2.24E-02 | regulation of epithelial cell differentiation | BTG1\|ALOX12\|TP63 |
| 43170 | 2.56E-03 | 2.24E-02 | macromolecule metabolic process | CUL9\|SMARCAL1\|DIDO1\|BTG2\|BTG1\|EIF4A3\|MST1R\|HDAC8\|EGFR\|UBE2J1\|COP1\|CHAF1B\|CHAF1A\|SUMO1\|OS9\|SIN3A\|DPH2\|MAGOH\|TCEB3\|TP53BP1\|RANBP2\|PDIA2\|MAP3K3\|NOP14\|UBE2I\|WNT5A\|ARNT\|RPA2\|BRAF\|ETV1\|FOS\|POU2F2\|PAX2\|MTOR\|JMJD6\|CLK2\|AR\|CAMK4\|SCG5\|MDM2\|TRIB3\|ATM\|ROR2\|TP53\|STK40\|PPIC\|TP73\|HSPA1A |
| 9719 | 2.70E-03 | 2.35E-02 | response to endogenous stimulus | PIAS3\|AR\|BTG2\|HHEX\|BTG1\|WNT5A\|TRIB3\|BRAF\|FOS\|SLC18A2\|MTOR |
| 6139 | 2.85E-03 | 2.46E-02 | nucleobase, nucleoside, nucleotide and nucleic acid metabolic process | SMARCAL1\|DIDO1\|BTG2\|EIF4A3\|CHAF1B\|CHAF1A\|SUMO1\|SIN3A\|MAGOH\|TCEB3\|TP53BP1\|NOP14\|ARNT\|RPA2\|ETV1\|FOS\|POU2F2\|PAX2\|JMJD6\|AR\|TP53I3\|ATM\|ROR2\|TP53\|TP73\|HSPA1A |
| 51322 | 2.96E-03 | 2.55E-02 | anaphase | SKA1\|SKA2 |
| 6351 | 2.98E-03 | 2.56E-02 | transcription, DNA-dependent | AR\|TCEB3\|ARNT\|RPA2\|ETV1\|POU2F2\|TP53\|PAX2 |
| 6401 | 3.04E-03 | 2.58E-02 | RNA catabolic process | EIF4A3\|MAGOH\|ATM\|HSPA1A |
| 6810 | 3.04E-03 | 2.58E-02 | transport | STEAP3\|RAB3A\|EIF4A3\|CPLX1\|ARNTL\|HHEX\|YWHAQ\|CUX1\|SV2A\|MAGOH\|SIX3\|SH3BP4\|SLC17A7\|MYH10\|SLC18A2\|TMED4\|SLC18A3\|RANBP2\|SYT4\|SYT1\|SLC32A1\|PDX1\|ARNT\|JMJD6\|EHD1\|AR\|MYO5B\|SCG5\|RIN3\|KCNK1\|TP53\|LLGL1\|VAMP2\|TP73 |
| 7399 | 3.11E-03 | 2.62E-02 | nervous system development | TFAP2A\|TCF7L2\|BTG2\|RAB3A\|FZD2\|FZD5\|WNT5A\|PAX6\|FOS\|NPAS2\|PAX2\|EGFR\|HHEX\|SIX3\|ATM\|ROR2\|TP53\|TP63\|TP73 |
| 32774 | 3.24E-03 | 2.72E-02 | RNA biosynthetic process | AR\|TCEB3\|ARNT\|RPA2\|ETV1\|POU2F2\|TP53\|PAX2 |
| 8283 | 3.30E-03 | 2.75E-02 | cell proliferation | AR\|TCF7L2\|HHEX\|BAD\|WNT5A\|BHLHE41\|MDM4\|TP53\|TP63\|EGFR |
| 7346 | 3.33E-03 | 2.77E-02 | regulation of mitotic cell cycle | CUL9\|NUSAP1\|MDM2\|ATM\|TP53\|EGFR |
| 6606 | 3.35E-03 | 2.77E-02 | protein import into nucleus | RANBP2\|SIX3\|TP53\|ARNTL |
| 35295 | 3.38E-03 | 2.78E-02 | tube development | TFAP2A\|AR\|HHEX\|RAB3A\|RBM15\|WNT5A\|TP63\|JMJD6 |
| 1763 | 3.39E-03 | 2.78E-02 | morphogenesis of a branching structure | AR\|HHEX\|RBM15\|WNT5A\|TP63 |
| 48869 | 3.42E-03 | 2.78E-02 | cellular developmental process | BTG2\|RAB3A\|BHLHE41\|EGFR\|HHEX\|SIX3\|TP63\|WWOX\|TCF7L2\|FZD2\|FZD5\|WNT5A\|PDX1\|ARNT\|MITF\|PAX6\|PAX2\|MTOR\|JMJD6\|AR\|PAX8\|ATM\|ROR2\|TP53\|TP73 |
| 7548 | 3.42E-03 | 2.78E-02 | sex differentiation | AR\|TCF7L2\|WNT5A\|NRIP1\|ROR2\|TP63 |
| 31399 | 3.49E-03 | 2.81E-02 | regulation of protein modification process | PIAS4\|PIAS3\|WNT5A\|ARNT\|TRIB3\|TP53\|EGFR\|MTOR\|TP73 |
| 45786 | 3.52E-03 | 2.81E-02 | negative regulation of cell cycle | TCF7L2\|SESN1\|ATM\|TP53\|EGFR\|TP73 |
| 45862 | 3.55E-03 | 2.81E-02 | positive regulation of proteolysis | SUMO1\|MDM2\|TRIB1 |
| 7269 | 3.55E-03 | 2.81E-02 | neurotransmitter secretion | SYT4\|RAB3A\|SYT1 |
| 30858 | 3.60E-03 | 2.81E-02 | positive regulation of epithelial cell differentiation | BTG1\|ALOX12 |
| 35020 | 3.60E-03 | 2.81E-02 | regulation of Rac protein signal transduction | CRK\|MTOR |
| 43516 | 3.60E-03 | 2.81E-02 | regulation of DNA damage response, signal transduction by p53 class mediator | MDM2\|ATM |
| 60742 | 3.60E-03 | 2.81E-02 | epithelial cell differentiation involved in prostate gland development | AR\|TP63 |
| 30182 | 3.81E-03 | 2.97E-02 | neuron differentiation | BTG2\|RAB3A\|FZD2\|FZD5\|WNT5A\|PAX6\|ROR2\|PAX2\|EGFR\|TP73 |
| 51170 | 3.87E-03 | 2.97E-02 | nuclear import | RANBP2\|SIX3\|TP53\|ARNTL |
| 35270 | 3.87E-03 | 2.97E-02 | endocrine system development | HHEX\|PAX8\|WNT5A\|PDX1 |
| 7417 | 3.87E-03 | 2.97E-02 | central nervous system development | HHEX\|WNT5A\|SIX3\|ATM\|PAX6\|ROR2\|TP53\|NPAS2\|EGFR\|TP73 |
| 48646 | 4.02E-03 | 3.06E-02 | anatomical structure formation involved in morphogenesis | TFAP2A\|AR\|HHEX\|RBM15\|WNT5A\|ATM\|ROR2\|TP53\|TP63 |
| 45926 | 4.03E-03 | 3.06E-02 | negative regulation of growth | BTG1\|TP53\|TP63\|TP73\|HSPA1A |
| 46777 | 4.24E-03 | 3.20E-02 | protein amino acid autophosphorylation | MAP3K3\|ATM\|EGFR\|MTOR |
| 22612 | 4.24E-03 | 3.20E-02 | gland morphogenesis | AR\|WNT5A\|TP63\|EGFR |
| 30335 | 4.31E-03 | 3.23E-02 | positive regulation of cell migration | WNT5A\|ARNT\|ALOX12\|ROR2\|EGFR |
| 165 | 4.61E-03 | 3.45E-02 | MAPKKK cascade | MAP3K3\|WNT5A\|BRAF\|ROR2\|TRIB1\|EGFR |
| 80135 | 4.75E-03 | 3.50E-02 | regulation of cellular response to stress | WNT5A\|MDM2\|ATM\|MTOR\|TP73 |
| 956 | 4.76E-03 | 3.50E-02 | nuclear-transcribed mRNA catabolic process | EIF4A3\|MAGOH\|ATM |
| 1756 | 4.76E-03 | 3.50E-02 | somitogenesis | ATM\|ROR2\|TP53 |
| 31016 | 4.76E-03 | 3.50E-02 | pancreas development | TCF7L2\|HHEX\|PDX1 |
| 8284 | 4.96E-03 | 3.62E-02 | positive regulation of cell proliferation | WNT5A\|MDM2\|PDX1\|ARNT\|ALOX12\|MDM4\|MST1R\|TP63\|EGFR\|MTOR |
| 71214 | 5.05E-03 | 3.62E-02 | cellular response to abiotic stimulus | ATM\|TP53 |
| 32350 | 5.05E-03 | 3.62E-02 | regulation of hormone metabolic process | TCF7L2\|ARNT |
| 23061 | 5.06E-03 | 3.62E-02 | signal release | SYT4\|RAB3A\|SYT1\|PDX1 |
| 7059 | 5.06E-03 | 3.62E-02 | chromosome segregation | UBE2I\|NUSAP1\|SKA1\|SKA2 |
| 3001 | 5.06E-03 | 3.62E-02 | generation of a signal involved in cell-cell signaling | SYT4\|RAB3A\|SYT1\|PDX1 |
| 23046 | 5.11E-03 | 3.63E-02 | signaling process | RAB3A\|MST1R\|CPLX1\|EGFR\|YWHAQ\|SLC17A7\|SYT4\|MAP3K3\|FZD2\|FZD5\|SYT1\|BAD\|WNT5A\|PDX1\|BRAF\|FOS\|RANGAP1\|MTOR\|AR\|CAMK4\|FAS\|RIN3\|ATM\|ROR2\|TRIB1\|CLOCK\|TP53\|CRK\|PPIC |
| 23060 | 5.11E-03 | 3.63E-02 | signal transmission | RAB3A\|MST1R\|CPLX1\|EGFR\|YWHAQ\|SLC17A7\|SYT4\|MAP3K3\|FZD2\|FZD5\|SYT1\|BAD\|WNT5A\|PDX1\|BRAF\|FOS\|RANGAP1\|MTOR\|AR\|CAMK4\|FAS\|RIN3\|ATM\|ROR2\|TRIB1\|CLOCK\|TP53\|CRK\|PPIC |
| 51128 | 5.22E-03 | 3.69E-02 | regulation of cellular component organization | CUL9\|RAB3A\|SUMO1\|NUSAP1\|ATM\|TP53\|MYH10\|SKA1\|MTOR\|SKA2\|HSPA1A |
| 48732 | 5.51E-03 | 3.88E-02 | gland development | AR\|HHEX\|PAX8\|WNT5A\|TP63\|EGFR |
| 45165 | 5.56E-03 | 3.90E-02 | cell fate commitment | TCF7L2\|SIX3\|MITF\|PAX6\|TP53 |
| 51272 | 5.73E-03 | 3.99E-02 | positive regulation of cellular component movement | WNT5A\|ARNT\|ALOX12\|ROR2\|EGFR |
| 40017 | 5.73E-03 | 3.99E-02 | positive regulation of locomotion | WNT5A\|ARNT\|ALOX12\|ROR2\|EGFR |
| 30183 | 5.81E-03 | 4.01E-02 | B cell differentiation | HHEX\|ATM\|TP53 |
| 61053 | 5.81E-03 | 4.01E-02 | somite development | ATM\|ROR2\|TP53 |
| 30878 | 5.86E-03 | 4.01E-02 | thyroid gland development | HHEX\|PAX8 |
| 51602 | 5.86E-03 | 4.01E-02 | response to electrical stimulus | BTG2\|RAB3A |
| 50896 | 5.94E-03 | 4.05E-02 | response to stimulus | BTG2\|RAB3A\|BTG1\|ALOX12\|MST1R\|NPAS2\|EGFR\|DEC1\|CHAF1B\|HHEX\|CHAF1A\|SUMO1\|OS9\|SESN1\|TP53BP1\|TP63\|SLC18A2\|PDIA2\|PIAS3\|TCF7L2\|SYT1\|WNT5A\|ARNT\|RPA2\|BRAF\|PAX6\|FOS\|POU2F2\|ETV5\|MTOR\|AR\|BHLHE40\|FAS\|TRIB3\|ATM\|ROR2\|TRIB1\|CALM1\|KCNK1\|CLOCK\|TP53\|TP73\|HSPA1A |
| 30111 | 5.97E-03 | 4.05E-02 | regulation of Wnt receptor signaling pathway | WWOX\|HHEX\|WNT5A\|SIX3 |
| 71103 | 6.09E-03 | 4.11E-02 | DNA conformation change | CHAF1B\|HHEX\|CHAF1A\|AIFM2\|NUSAP1 |
| 10648 | 6.11E-03 | 4.11E-02 | negative regulation of cell communication | WWOX\|HHEX\|WNT5A\|SIX3\|MDM2\|TRIB1\|TP53\|MTOR |
| 35467 | 6.30E-03 | 4.22E-02 | negative regulation of signaling pathway | WWOX\|HHEX\|WNT5A\|SIX3\|MDM2\|TRIB1\|TP53 |
| 6916 | 6.38E-03 | 4.26E-02 | anti-apoptosis | TCF7L2\|FAS\|ALOX12\|BRAF\|TP63\|HSPA1A |
| 43412 | 6.43E-03 | 4.28E-02 | macromolecule modification | MAP3K3\|BTG2\|UBE2I\|BTG1\|WNT5A\|BRAF\|MST1R\|FOS\|HDAC8\|EGFR\|MTOR\|JMJD6\|UBE2J1\|CLK2\|SUMO1\|OS9\|DPH2\|CAMK4\|MDM2\|TRIB3\|ATM\|ROR2\|STK40 |
| 35239 | 6.69E-03 | 4.36E-02 | tube morphogenesis | TFAP2A\|AR\|HHEX\|RBM15\|WNT5A\|TP63 |
| 9628 | 6.71E-03 | 4.36E-02 | response to abiotic stimulus | DEC1\|BTG2\|RAB3A\|BHLHE40\|ATM\|FOS\|CLOCK\|TP53\|EGFR |
| 42771 | 6.72E-03 | 4.36E-02 | DNA damage response, signal transduction by p53 class mediator resulting in induction of apoptosis | TP53\|TP63 |
| 60444 | 6.72E-03 | 4.36E-02 | branching involved in mammary gland duct morphogenesis | AR\|WNT5A |
| 32092 | 6.72E-03 | 4.36E-02 | positive regulation of protein binding | TCF7L2\|TRIB3 |
| 51028 | 6.73E-03 | 4.36E-02 | mRNA transport | RANBP2\|HHEX\|EIF4A3\|MAGOH |
| 48754 | 6.73E-03 | 4.36E-02 | branching morphogenesis of a tube | AR\|HHEX\|RBM15\|WNT5A |
| 1936 | 6.98E-03 | 4.49E-02 | regulation of endothelial cell proliferation | WNT5A\|ARNT\|MTOR |
| 43523 | 6.99E-03 | 4.49E-02 | regulation of neuron apoptosis | BRAF\|ATM\|TP53\|TP73 |
| 48598 | 7.02E-03 | 4.50E-02 | embryonic morphogenesis | TFAP2A\|TCF7L2\|HHEX\|WNT5A\|PDX1\|ROR2\|TP53\|TP63 |
| 6464 | 7.12E-03 | 4.54E-02 | protein modification process | MAP3K3\|BTG2\|UBE2I\|BTG1\|WNT5A\|BRAF\|MST1R\|HDAC8\|EGFR\|MTOR\|JMJD6\|UBE2J1\|CLK2\|SUMO1\|OS9\|DPH2\|CAMK4\|MDM2\|TRIB3\|ATM\|ROR2\|STK40 |
| 6913 | 7.26E-03 | 4.61E-02 | nucleocytoplasmic transport | RANBP2\|HHEX\|SIX3\|TP53\|ARNTL |
| 51169 | 7.47E-03 | 4.73E-02 | nuclear transport | RANBP2\|HHEX\|SIX3\|TP53\|ARNTL |
| 42770 | 7.54E-03 | 4.76E-02 | DNA damage response, signal transduction | ATM\|TP53\|TP63\|TP73 |
| 45599 | 7.64E-03 | 4.76E-02 | negative regulation of fat cell differentiation | WNT5A\|TRIB3 |
| 70972 | 7.64E-03 | 4.76E-02 | protein localization in endoplasmic reticulum | PDIA2\|OS9 |
| 30947 | 7.64E-03 | 4.76E-02 | regulation of vascular endothelial growth factor receptor signaling pathway | HHEX\|ARNT |
| 48731 | 7.67E-03 | 4.76E-02 | system development | BTG2\|RAB3A\|BHLHE41\|NPAS2\|EGFR\|SIX6\|HHEX\|SIX3\|NRIP1\|TP63\|TFAP2A\|WWOX\|TCF7L2\|FZD2\|RBM15\|FZD5\|WNT5A\|PDX1\|ARNT\|MITF\|BRAF\|PAX6\|FOS\|PAX2\|JMJD6\|AR\|PAX8\|ATM\|ROR2\|TP53\|TP73 |
| 1654 | 7.68E-03 | 4.76E-02 | eye development | WNT5A\|SIX3\|MITF\|PAX6\|JMJD6 |
| 30098 | 8.12E-03 | 4.79E-02 | lymphocyte differentiation | HHEX\|ATM\|TP53\|JMJD6 |
| 70077 | 8.32E-03 | 4.79E-02 | histone arginine demethylation | JMJD6 |
| 70079 | 8.32E-03 | 4.79E-02 | histone H4-R3 demethylation | JMJD6 |
| 70078 | 8.32E-03 | 4.79E-02 | histone H3-R2 demethylation | JMJD6 |
| 17126 | 8.32E-03 | 4.79E-02 | nucleologenesis | RPA2 |
| 17185 | 8.32E-03 | 4.79E-02 | peptidyl-lysine hydroxylation | JMJD6 |
| 21506 | 8.32E-03 | 4.79E-02 | anterior neuropore closure | TFAP2A |
| 42137 | 8.32E-03 | 4.79E-02 | sequestering of neurotransmitter | SLC17A7 |
| 21995 | 8.32E-03 | 4.79E-02 | neuropore closure | TFAP2A |
| 18395 | 8.32E-03 | 4.79E-02 | peptidyl-lysine hydroxylation to 5-hydroxy-L-lysine | JMJD6 |
| 43610 | 8.32E-03 | 4.79E-02 | regulation of carbohydrate utilization | MTOR |
| 19102 | 8.32E-03 | 4.79E-02 | male somatic sex determination | AR |
| 2741 | 8.32E-03 | 4.79E-02 | positive regulation of cytokine secretion involved in immune response | WNT5A |
| 31630 | 8.32E-03 | 4.79E-02 | regulation of synaptic vesicle fusion to presynaptic membrane | RAB3A |
| 60316 | 8.32E-03 | 4.79E-02 | positive regulation of ryanodine-sensitive calcium-release channel activity | CALM1 |
| 60520 | 8.32E-03 | 4.79E-02 | activation of prostate induction by androgen receptor signaling pathway | AR |
| 60529 | 8.32E-03 | 4.79E-02 | squamous basal epithelial stem cell differentiation involved in prostate gland acinus development | TP63 |
| 61009 | 8.32E-03 | 4.79E-02 | bile duct development | HHEX |
| 61011 | 8.32E-03 | 4.79E-02 | hepatic duct development | HHEX |
| 48790 | 8.32E-03 | 4.79E-02 | maintenance of presynaptic active zone structure | RAB3A |
| 45023 | 8.32E-03 | 4.79E-02 | G0 to G1 transition | MDM4 |
| 6468 | 8.55E-03 | 4.91E-02 | protein amino acid phosphorylation | MAP3K3\|CAMK4\|WNT5A\|TRIB3\|BRAF\|ATM\|ROR2\|MST1R\|STK40\|EGFR\|MTOR\|CLK2 |
| 1558 | 8.59E-03 | 4.91E-02 | regulation of cell growth | BTG1\|ALOX12\|TP53\|TP63\|TP73\|HSPA1A |
| 10149 | 8.61E-03 | 4.91E-02 | senescence | ATM\|TP53 |
| 33500 | 8.76E-03 | 4.96E-02 | carbohydrate homeostasis | TCF7L2\|BAD\|PDX1 |
| 42593 | 8.76E-03 | 4.96E-02 | glucose homeostasis | TCF7L2\|BAD\|PDX1 |
| 43405 | 8.80E-03 | 4.97E-02 | regulation of MAP kinase activity | WNT5A\|TRIB3\|TRIB1\|EGFR\|TP73 |

(B)

| **GO-ID** | **p-value** | **corr p-value** | **Description** | **Genes in test set** |
| --- | --- | --- | --- | --- |
| 7059 | 7.09E-09 | 1.00E-05 | chromosome segregation | STAG1\|STAG2\|CDCA5\|RAD21\|PDS5B\|BRCA1\|SMC3\|SMC1A\|REC8 |
| 7062 | 3.18E-08 | 2.24E-05 | sister chromatid cohesion | STAG2\|PDS5B\|SMC3\|SMC1A\|REC8 |
| 30258 | 3.78E-07 | 1.78E-04 | lipid modification | PIK3CA\|HACL1\|PIK3C3\|PIK3R1\|ACADM\|HADH\|ACADS |
| 6104 | 1.40E-06 | 4.94E-04 | succinyl-CoA metabolic process | SUCLA2\|SUCLG2\|SUCLG1 |
| 48523 | 2.70E-06 | 7.02E-04 | negative regulation of cellular process | BECN1\|CTBP2\|CTBP1\|FMR1\|PDS5B\|BRCA1\|SMC3\|HDAC8\|TOB1\|PTPRF\|PPM1G\|FXR1\|RXRA\|SUFU\|NRIP2\|SOX6\|ZBP1\|PRKCI\|PTPN11\|SMC1A\|NGF\|RUNX1\|SOD1\|STAG2\|ZEB1\|PIK3CA\|TLR9\|SPRY2\|PPARA\|FGFR1\|FOXA2 |
| 32875 | 3.49E-06 | 7.02E-04 | regulation of DNA endoreduplication | STAG2\|SMC3\|SMC1A |
| 32876 | 3.49E-06 | 7.02E-04 | negative regulation of DNA endoreduplication | STAG2\|SMC3\|SMC1A |
| 48519 | 1.78E-05 | 2.99E-03 | negative regulation of biological process | BECN1\|CTBP2\|CTBP1\|FMR1\|PDS5B\|BRCA1\|SMC3\|HDAC8\|TOB1\|PTPRF\|PPM1G\|FXR1\|RXRA\|SUFU\|NRIP2\|SOX6\|ZBP1\|PRKCI\|PTPN11\|SMC1A\|NGF\|RUNX1\|SOD1\|STAG2\|ZEB1\|PIK3CA\|TLR9\|SPRY2\|PPARA\|FGFR1\|FOXA2 |
| 10558 | 1.91E-05 | 2.99E-03 | negative regulation of macromolecule biosynthetic process | ZBP1\|CTBP1\|FMR1\|BRCA1\|SMC3\|SMC1A\|HDAC8\|FXR1\|STAG2\|ZEB1\|RXRA\|SUFU\|NRIP2\|SOX6\|PPARA |
| 31327 | 2.70E-05 | 3.81E-03 | negative regulation of cellular biosynthetic process | ZBP1\|CTBP1\|FMR1\|BRCA1\|SMC3\|SMC1A\|HDAC8\|FXR1\|STAG2\|ZEB1\|RXRA\|SUFU\|NRIP2\|SOX6\|PPARA |
| 9890 | 3.26E-05 | 4.15E-03 | negative regulation of biosynthetic process | ZBP1\|CTBP1\|FMR1\|BRCA1\|SMC3\|SMC1A\|HDAC8\|FXR1\|STAG2\|ZEB1\|RXRA\|SUFU\|NRIP2\|SOX6\|PPARA |
| 279 | 4.04E-05 | 4.15E-03 | M phase | STAG1\|STAG2\|CDCA5\|RAD21\|CCNG2\|PDS5B\|SMC3\|PDS5A\|SMC1A\|REC8\|SMC1B |
| 280 | 4.12E-05 | 4.15E-03 | nuclear division | STAG1\|STAG2\|CDCA5\|RAD21\|CCNG2\|PDS5B\|SMC3\|PDS5A\|SMC1A |
| 7067 | 4.12E-05 | 4.15E-03 | mitosis | STAG1\|STAG2\|CDCA5\|RAD21\|CCNG2\|PDS5B\|SMC3\|PDS5A\|SMC1A |
| 87 | 5.20E-05 | 4.88E-03 | M phase of mitotic cell cycle | STAG1\|STAG2\|CDCA5\|RAD21\|CCNG2\|PDS5B\|SMC3\|PDS5A\|SMC1A |
| 48285 | 5.54E-05 | 4.88E-03 | organelle fission | STAG1\|STAG2\|CDCA5\|RAD21\|CCNG2\|PDS5B\|SMC3\|PDS5A\|SMC1A |
| 50794 | 6.86E-05 | 5.49E-03 | regulation of cellular process | FMR1\|BRCA1\|SMC3\|CYR61\|PTPRF\|ARL5B\|SUFU\|CWC22\|SOX6\|ZBP1\|TLE3\|PRKCI\|TLE2\|SOX13\|PPP2R5B\|FRS2\|SMC1A\|NGF\|RUNX1\|DDX19B\|ZEB1\|PIK3CA\|TBC1D5\|TLR9\|ULK2\|STRAP\|PIK3C3\|PPARA\|ARF5\|RBPJL\|BECN1\|CTBP2\|CAB39\|CTBP1\|DAPP1\|TPTE\|PIK3R1\|PDS5B\|HDAC8\|TOB1\|PPM1G\|FXR1\|RXRB\|RAP1A\|RXRA\|EGFLAM\|RAD21\|NRIP2\|ZKSCAN3\|ZSCAN12\|PLCG1\|PTPN11\|KLF3\|QKI\|REC8\|SOD1\|GOLPH3\|GPR143\|STAG2\|CD5\|CCNG2\|SPRY2\|FGFR1\|FOXA2 |
| 46834 | 7.40E-05 | 5.49E-03 | lipid phosphorylation | PIK3CA\|PIK3C3\|PIK3R1 |
| 46854 | 7.40E-05 | 5.49E-03 | phosphoinositide phosphorylation | PIK3CA\|PIK3C3\|PIK3R1 |
| 51327 | 8.80E-05 | 5.91E-03 | M phase of meiotic cell cycle | STAG2\|RAD21\|SMC3\|SMC1A\|REC8\|SMC1B |
| 7126 | 8.80E-05 | 5.91E-03 | meiosis | STAG2\|RAD21\|SMC3\|SMC1A\|REC8\|SMC1B |
| 51321 | 9.29E-05 | 5.95E-03 | meiotic cell cycle | STAG2\|RAD21\|SMC3\|SMC1A\|REC8\|SMC1B |
| 44248 | 1.15E-04 | 6.76E-03 | cellular catabolic process | BECN1\|USP43\|EDEM1\|AMBRA1\|ATG14\|SOD1\|SUCLA2\|IVD\|SUCLG2\|SUCLG1\|HACL1\|PLCG1\|ACADM\|HADH\|ACADS\|AASS |
| 10551 | 1.15E-04 | 6.76E-03 | regulation of gene-specific transcription from RNA polymerase II promoter | RXRA\|RAD21\|SUFU\|TLR9\|BRCA1\|PPARA\|FOXA2 |
| 44237 | 1.33E-04 | 7.49E-03 | cellular metabolic process | WDR48\|ETFA\|ETFB\|BRCA1\|SMC3\|PTPRF\|RNF19A\|CWC22\|ACADM\|HADH\|ACADS\|AASS\|ZBP1\|PRKCI\|USP43\|UBE2E2\|FRS2\|ATG14\|SMC1A\|SRRM1\|NEIL2\|PIK3CA\|IVD\|TLR9\|ULK2\|SUCLG2\|STRAP\|SUCLG1\|PIK3C3\|PPARA\|BECN1\|CTBP1\|AK1\|PIK3R4\|DAPP1\|TPTE\|PIK3R1\|AMBRA1\|HDAC8\|HSD17B10\|PPM1G\|EGFLAM\|RAD21\|HACL1\|PLCG1\|SLC35A2\|SLC35A3\|EDEM1\|PTPN11\|QKI\|REC8\|SOD1\|SUCLA2\|FGFR1 |
| 19395 | 1.66E-04 | 8.65E-03 | fatty acid oxidation | HACL1\|ACADM\|HADH\|ACADS |
| 34440 | 1.66E-04 | 8.65E-03 | lipid oxidation | HACL1\|ACADM\|HADH\|ACADS |
| 46395 | 1.96E-04 | 8.89E-03 | carboxylic acid catabolic process | IVD\|HACL1\|ACADM\|HADH\|ACADS\|AASS |
| 16054 | 1.96E-04 | 8.89E-03 | organic acid catabolic process | IVD\|HACL1\|ACADM\|HADH\|ACADS\|AASS |
| 50789 | 1.99E-04 | 8.89E-03 | regulation of biological process | FMR1\|BRCA1\|SMC3\|CYR61\|PTPRF\|ARL5B\|SUFU\|CWC22\|SOX6\|ZBP1\|TLE3\|PRKCI\|TLE2\|SOX13\|PPP2R5B\|UBE2E2\|FRS2\|SMC1A\|NGF\|RUNX1\|DDX19B\|ZEB1\|PIK3CA\|TBC1D5\|TLR9\|ULK2\|STRAP\|PIK3C3\|PPARA\|ARF5\|RBPJL\|BECN1\|CTBP2\|CAB39\|CTBP1\|DAPP1\|TPTE\|PIK3R1\|PDS5B\|HDAC8\|TOB1\|PPM1G\|FXR1\|RXRB\|RAP1A\|RXRA\|EGFLAM\|RAD21\|NRIP2\|ZKSCAN3\|ZSCAN12\|PLCG1\|PTPN11\|KLF3\|QKI\|REC8\|SOD1\|GOLPH3\|GPR143\|STAG2\|CD5\|CCNG2\|SPRY2\|FGFR1\|FOXA2 |
| 10948 | 2.02E-04 | 8.89E-03 | negative regulation of cell cycle process | STAG2\|BRCA1\|SMC3\|SMC1A |
| 9062 | 2.02E-04 | 8.89E-03 | fatty acid catabolic process | HACL1\|ACADM\|HADH\|ACADS |
| 90329 | 2.66E-04 | 1.08E-02 | regulation of DNA-dependent DNA replication | STAG2\|SMC3\|SMC1A |
| 35265 | 2.66E-04 | 1.08E-02 | organ growth | RXRA\|SPRY2\|PTPN11 |
| 22403 | 2.69E-04 | 1.08E-02 | cell cycle phase | STAG1\|STAG2\|CDCA5\|RAD21\|CCNG2\|PDS5B\|SMC3\|PDS5A\|SMC1A\|REC8\|SMC1B |
| 44242 | 3.13E-04 | 1.22E-02 | cellular lipid catabolic process | HACL1\|PLCG1\|ACADM\|HADH\|ACADS |
| 8152 | 3.38E-04 | 1.29E-02 | metabolic process | WDR48\|ETFA\|ETFB\|BRCA1\|SMC3\|PTPRF\|RNF19A\|SUFU\|CWC22\|ACADM\|HADH\|ACADS\|AASS\|ZBP1\|PRKCI\|USP43\|UBE2E2\|FRS2\|ATG14\|SMC1A\|NGF\|SRRM1\|NEIL2\|PIK3CA\|IVD\|TLR9\|ULK2\|SUCLG2\|STRAP\|SUCLG1\|PIK3C3\|PPARA\|BECN1\|CTBP2\|CTBP1\|AK1\|PIK3R4\|DAPP1\|TPTE\|PIK3R1\|AMBRA1\|HDAC8\|HSD17B10\|PPM1G\|RXRA\|EGFLAM\|RAD21\|NRIP2\|HACL1\|PLCG1\|SLC35A2\|SLC35A3\|EDEM1\|PTPN11\|QKI\|REC8\|SOD1\|GPR143\|SUCLA2\|FGFR1 |
| 9892 | 3.89E-04 | 1.44E-02 | negative regulation of metabolic process | ZBP1\|CTBP1\|FMR1\|BRCA1\|SMC3\|SMC1A\|HDAC8\|SOD1\|FXR1\|STAG2\|ZEB1\|RXRA\|SUFU\|NRIP2\|SOX6\|PPARA |
| 51301 | 4.05E-04 | 1.46E-02 | cell division | STAG1\|STAG2\|CDCA5\|RAD21\|CCNG2\|PDS5B\|SMC3\|PDS5A\|SMC1A |
| 31324 | 4.39E-04 | 1.52E-02 | negative regulation of cellular metabolic process | ZBP1\|CTBP1\|FMR1\|BRCA1\|SMC3\|SMC1A\|HDAC8\|FXR1\|STAG2\|ZEB1\|RXRA\|SUFU\|NRIP2\|SOX6\|PPARA |
| 6099 | 4.92E-04 | 1.52E-02 | tricarboxylic acid cycle | SUCLA2\|SUCLG2\|SUCLG1 |
| 51387 | 4.97E-04 | 1.52E-02 | negative regulation of nerve growth factor receptor signaling pathway | SPRY2\|PTPRF |
| 7064 | 4.97E-04 | 1.52E-02 | mitotic sister chromatid cohesion | PDS5B\|SMC1A |
| 15780 | 4.97E-04 | 1.52E-02 | nucleotide-sugar transport | SLC35A2\|SLC35A3 |
| 15781 | 4.97E-04 | 1.52E-02 | pyrimidine nucleotide-sugar transport | SLC35A2\|SLC35A3 |
| 90004 | 4.97E-04 | 1.52E-02 | positive regulation of establishment of protein localization in plasma membrane | PRKCI\|PIK3R1 |
| 10605 | 5.42E-04 | 1.63E-02 | negative regulation of macromolecule metabolic process | ZBP1\|CTBP1\|FMR1\|BRCA1\|SMC3\|SMC1A\|HDAC8\|FXR1\|STAG2\|ZEB1\|RXRA\|SUFU\|NRIP2\|SOX6\|PPARA |
| 46356 | 5.63E-04 | 1.65E-02 | acetyl-CoA catabolic process | SUCLA2\|SUCLG2\|SUCLG1 |
| 45934 | 5.90E-04 | 1.70E-02 | negative regulation of nucleobase, nucleoside, nucleotide and nucleic acid metabolic process | STAG2\|ZEB1\|RXRA\|CTBP1\|SUFU\|NRIP2\|BRCA1\|SMC3\|SOX6\|SMC1A\|PPARA\|HDAC8 |
| 51172 | 6.49E-04 | 1.83E-02 | negative regulation of nitrogen compound metabolic process | STAG2\|ZEB1\|RXRA\|CTBP1\|SUFU\|NRIP2\|BRCA1\|SMC3\|SOX6\|SMC1A\|PPARA\|HDAC8 |
| 51386 | 7.42E-04 | 2.05E-02 | regulation of nerve growth factor receptor signaling pathway | SPRY2\|PTPRF |
| 70201 | 7.87E-04 | 2.13E-02 | regulation of establishment of protein localization | PRKCI\|SUFU\|TLR9\|PTPN11\|PIK3R1\|REC8 |
| 32583 | 8.00E-04 | 2.13E-02 | regulation of gene-specific transcription | RXRA\|RAD21\|SUFU\|TLR9\|BRCA1\|PPARA\|FOXA2 |
| 22402 | 8.81E-04 | 2.25E-02 | cell cycle process | STAG1\|STAG2\|CDCA5\|RAD21\|CCNG2\|PDS5B\|SMC3\|PDS5A\|SMC1A\|REC8\|SMC1B\|PPM1G |
| 23046 | 9.01E-04 | 2.25E-02 | signaling process | RBPJL\|DAPP1\|TPTE\|PIK3R1\|BRCA1\|SMC3\|PTPRF\|ARL5B\|RAP1A\|SUFU\|PLCG1\|TLE3\|TLE2\|PPP2R5B\|FRS2\|PTPN11\|NGF\|QKI\|REC8\|SOD1\|GPR143\|PIK3CA\|TLR9\|ULK2\|SPRY2\|PIK3C3\|ARF5\|FGFR1 |
| 23060 | 9.01E-04 | 2.25E-02 | signal transmission | RBPJL\|DAPP1\|TPTE\|PIK3R1\|BRCA1\|SMC3\|PTPRF\|ARL5B\|RAP1A\|SUFU\|PLCG1\|TLE3\|TLE2\|PPP2R5B\|FRS2\|PTPN11\|NGF\|QKI\|REC8\|SOD1\|GPR143\|PIK3CA\|TLR9\|ULK2\|SPRY2\|PIK3C3\|ARF5\|FGFR1 |
| 9109 | 9.10E-04 | 2.25E-02 | coenzyme catabolic process | SUCLA2\|SUCLG2\|SUCLG1 |
| 122 | 1.00E-03 | 2.43E-02 | negative regulation of transcription from RNA polymerase II promoter | ZEB1\|RXRA\|CTBP1\|SUFU\|NRIP2\|SOX6\|PPARA\|HDAC8 |
| 7185 | 1.03E-03 | 2.43E-02 | transmembrane receptor protein tyrosine phosphatase signaling pathway | FRS2\|PTPRF |
| 90003 | 1.03E-03 | 2.43E-02 | regulation of establishment of protein localization in plasma membrane | PRKCI\|PIK3R1 |
| 6793 | 1.07E-03 | 2.44E-02 | phosphorus metabolic process | PRKCI\|CTBP1\|PIK3R4\|DAPP1\|FRS2\|TPTE\|PTPN11\|PIK3R1\|PTPRF\|PPM1G\|SOD1\|PIK3CA\|TLR9\|ULK2\|PIK3C3\|FGFR1 |
| 6796 | 1.07E-03 | 2.44E-02 | phosphate metabolic process | PRKCI\|CTBP1\|PIK3R4\|DAPP1\|FRS2\|TPTE\|PTPN11\|PIK3R1\|PTPRF\|PPM1G\|SOD1\|PIK3CA\|TLR9\|ULK2\|PIK3C3\|FGFR1 |
| 6635 | 1.13E-03 | 2.52E-02 | fatty acid beta-oxidation | ACADM\|HADH\|ACADS |
| 6084 | 1.24E-03 | 2.74E-02 | acetyl-CoA metabolic process | SUCLA2\|SUCLG2\|SUCLG1 |
| 7165 | 1.29E-03 | 2.79E-02 | signal transduction | RBPJL\|DAPP1\|TPTE\|PIK3R1\|BRCA1\|SMC3\|ARL5B\|RAP1A\|SUFU\|PLCG1\|TLE3\|TLE2\|PPP2R5B\|FRS2\|PTPN11\|NGF\|SOD1\|GPR143\|PIK3CA\|TLR9\|ULK2\|SPRY2\|PIK3C3\|ARF5\|FGFR1 |
| 50872 | 1.37E-03 | 2.93E-02 | white fat cell differentiation | CTBP2\|CTBP1 |
| 65007 | 1.40E-03 | 2.94E-02 | biological regulation | FMR1\|BRCA1\|SMC3\|CYR61\|PTPRF\|ARL5B\|SUFU\|CWC22\|SOX6\|ZBP1\|TLE3\|PRKCI\|TLE2\|SOX13\|PPP2R5B\|UBE2E2\|FRS2\|SMC1A\|NGF\|RUNX1\|DDX19B\|ZEB1\|PIK3CA\|TBC1D5\|TLR9\|ULK2\|STRAP\|PIK3C3\|PPARA\|ARF5\|RBPJL\|BECN1\|CTBP2\|CAB39\|CTBP1\|DAPP1\|TPTE\|PIK3R1\|PDS5B\|HDAC8\|TOB1\|PPM1G\|FXR1\|RXRB\|RAP1A\|RXRA\|EGFLAM\|RAD21\|NRIP2\|ZKSCAN3\|ZSCAN12\|PLCG1\|PTPN11\|KLF3\|QKI\|REC8\|SOD1\|GOLPH3\|GPR143\|STAG2\|CD5\|CCNG2\|SPRY2\|FGFR1\|FOXA2 |
| 43687 | 1.42E-03 | 2.94E-02 | post-translational protein modification | PRKCI\|WDR48\|CTBP1\|PIK3R4\|UBE2E2\|DAPP1\|FRS2\|TPTE\|PTPN11\|BRCA1\|HDAC8\|PTPRF\|PPM1G\|SOD1\|EGFLAM\|PIK3CA\|TLR9\|ULK2\|FGFR1 |
| 32880 | 1.44E-03 | 2.94E-02 | regulation of protein localization | PRKCI\|SUFU\|TLR9\|PTPN11\|PIK3R1\|REC8 |
| 8285 | 1.54E-03 | 3.09E-02 | negative regulation of cell proliferation | BECN1\|ZEB1\|RXRA\|CTBP2\|CTBP1\|SPRY2\|PDS5B\|TOB1\|PTPRF |
| 278 | 1.56E-03 | 3.10E-02 | mitotic cell cycle | STAG1\|STAG2\|CDCA5\|RAD21\|CCNG2\|PDS5B\|SMC3\|PDS5A\|SMC1A |
| 6996 | 1.62E-03 | 3.18E-02 | organelle organization | BECN1\|PRKCI\|CDCA5\|PDS5B\|SMC3\|ATG14\|PDS5A\|SMC1A\|HDAC8\|REC8\|SMC1B\|SOD1\|STAG1\|GPR143\|STAG2\|RNF19A\|RAD21\|CCNG2\|SOX6\|FOXA2 |
| 51187 | 1.65E-03 | 3.18E-02 | cofactor catabolic process | SUCLA2\|SUCLG2\|SUCLG1 |
| 9060 | 1.80E-03 | 3.38E-02 | aerobic respiration | SUCLA2\|SUCLG2\|SUCLG1 |
| 8156 | 1.80E-03 | 3.38E-02 | negative regulation of DNA replication | STAG2\|SMC3\|SMC1A |
| 44255 | 1.83E-03 | 3.39E-02 | cellular lipid metabolic process | PIK3CA\|HACL1\|PIK3C3\|PTPN11\|PIK3R1\|PLCG1\|ACADM\|HADH\|PPARA\|ACADS\|QKI |
| 32868 | 1.86E-03 | 3.40E-02 | response to insulin stimulus | PRKCI\|RXRA\|PIK3R1\|PPARA\|FOXA2 |
| 22008 | 1.90E-03 | 3.44E-02 | neurogenesis | PRKCI\|RXRA\|ULK2\|FRS2\|PTPN11\|SOX6\|NGF\|PTPRF\|REC8\|FGFR1\|SOD1\|FOXA2 |
| 9056 | 1.96E-03 | 3.50E-02 | catabolic process | BECN1\|USP43\|EDEM1\|AMBRA1\|ATG14\|SOD1\|SUCLA2\|IVD\|SUCLG2\|SUCLG1\|HACL1\|PLCG1\|ACADM\|HADH\|ACADS\|AASS |
| 70 | 2.12E-03 | 3.74E-02 | mitotic sister chromatid segregation | CDCA5\|PDS5B\|SMC1A |
| 6105 | 2.18E-03 | 3.78E-02 | succinate metabolic process | SUCLA2\|SUCLG1 |
| 43434 | 2.20E-03 | 3.78E-02 | response to peptide hormone stimulus | PRKCI\|RXRA\|PIK3R1\|NGF\|PPARA\|FOXA2 |
| 17148 | 2.30E-03 | 3.85E-02 | negative regulation of translation | ZBP1\|FXR1\|FMR1 |
| 819 | 2.30E-03 | 3.85E-02 | sister chromatid segregation | CDCA5\|PDS5B\|SMC1A |
| 6357 | 2.47E-03 | 4.10E-02 | regulation of transcription from RNA polymerase II promoter | CTBP1\|BRCA1\|HDAC8\|RUNX1\|ZEB1\|RXRA\|RAD21\|SUFU\|NRIP2\|TLR9\|SOX6\|PPARA\|FOXA2 |
| 6470 | 2.53E-03 | 4.15E-02 | protein amino acid dephosphorylation | DAPP1\|TPTE\|PTPN11\|PTPRF\|PPM1G |
| 33081 | 2.66E-03 | 4.26E-02 | regulation of T cell differentiation in the thymus | ZEB1\|SOD1 |
| 60525 | 2.66E-03 | 4.26E-02 | prostate glandular acinus development | RXRA\|FRS2 |
| 9719 | 2.70E-03 | 4.27E-02 | response to endogenous stimulus | PRKCI\|RXRA\|PTPN11\|PIK3R1\|BRCA1\|NGF\|PPARA\|ACADS\|SOD1\|FOXA2 |
| 42127 | 2.73E-03 | 4.27E-02 | regulation of cell proliferation | BECN1\|CTBP2\|CTBP1\|FRS2\|PDS5B\|BRCA1\|NGF\|TOB1\|PTPRF\|ZEB1\|RXRA\|CD5\|SPRY2\|FGFR1 |
| 45165 | 2.88E-03 | 4.41E-02 | cell fate commitment | SPRY2\|FRS2\|SOX6\|FGFR1\|FOXA2 |
| 16043 | 2.98E-03 | 4.53E-02 | cellular component organization | BECN1\|CDCA5\|PDS5B\|SMC3\|PDS5A\|HDAC8\|HSD17B10\|CYR61\|SNX1\|RXRA\|EGFLAM\|RNF19A\|RAD21\|SOX6\|AASS\|PRKCI\|PTPN11\|ATG14\|SMC1A\|NGF\|REC8\|SMC1B\|SOD1\|STAG1\|GPR143\|STAG2\|CCNG2\|ULK2\|FGFR1\|FOXA2 |
| 23034 | 3.27E-03 | 4.85E-02 | intracellular signaling pathway | PRKCI\|FRS2\|PTPN11\|PIK3R1\|BRCA1\|SMC1A\|NGF\|SOD1\|STAG1\|ARL5B\|RAP1A\|RXRA\|PIK3CA\|TLR9\|PIK3C3\|ARF5\|FGFR1 |

**Supplementary Figure 1.** Hierarchical clustering and corresponding heatmaps for the three groups of potential biomarkers identified with the BioDiscML software. The groups consisted of 100 genes (A), 75 genes (B) or 50 genes (C). The confusion matrix for the classifications and corresponding accuracy are shown below each heat map.


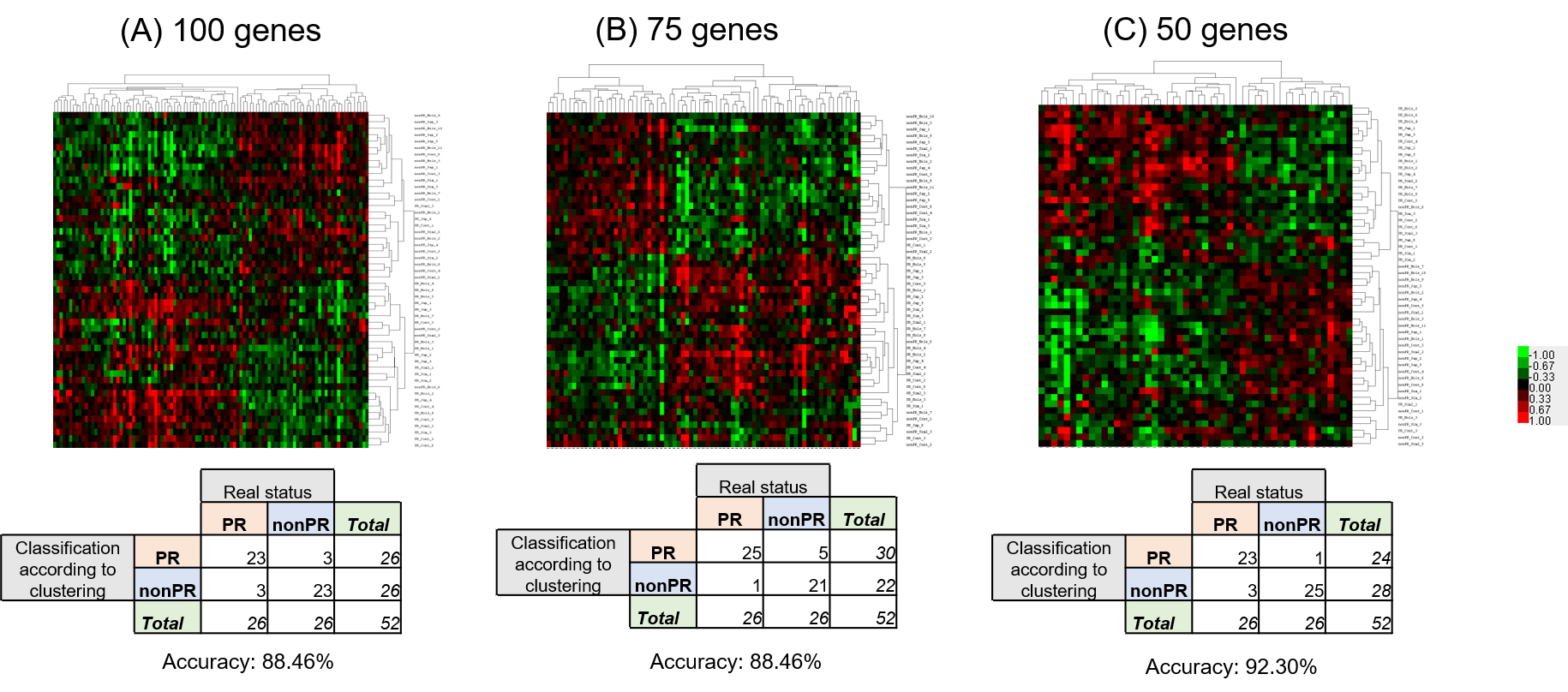


**Supplementary Figure 2.** Networks constructed with the biomarker genes (pink nodes), and inferred related genes (grey nodes), for the endometrial genes with increased (A) or decreased (B) expression in the animals that became pregnant. Genes (nodes) were connected if they were functionally associated (red edges), if their products interact (blue edges) or if they were in the same reaction within a pathway (green edges). Networks were generated with the Cytoscape software (V 3.7.2).

**Supplemental Figure 3.** Multidimensional Scaling analysis (MDS) of the 52 endometrial samples obtained at day 6-7 of the estrous cycle from different experiments/breeds. (A) MDS before batch correction (B) MDS after batch correction with ComBat.


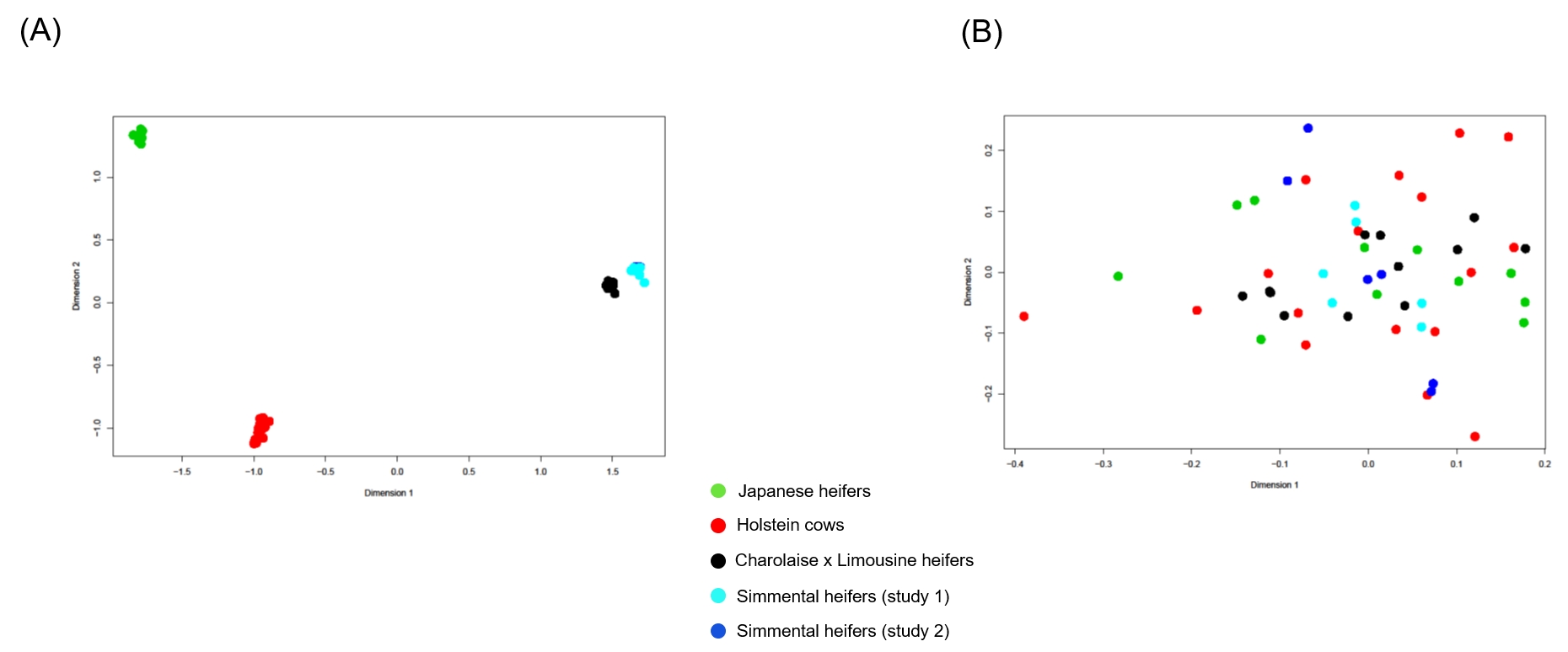


**Supplemental Figure 4.** (A) Average normalized expression for each of the 50 genes for samples classified as receptive (blue bars) or not (red bars) for each breed. (B) Difference in expression between receptive and non-receptive samples for each of the 50 genes from each breed. Holstein: green bars, Japanese black: orange bars, Charolais x Limousin: light blue bars, Simmental: purple bars.


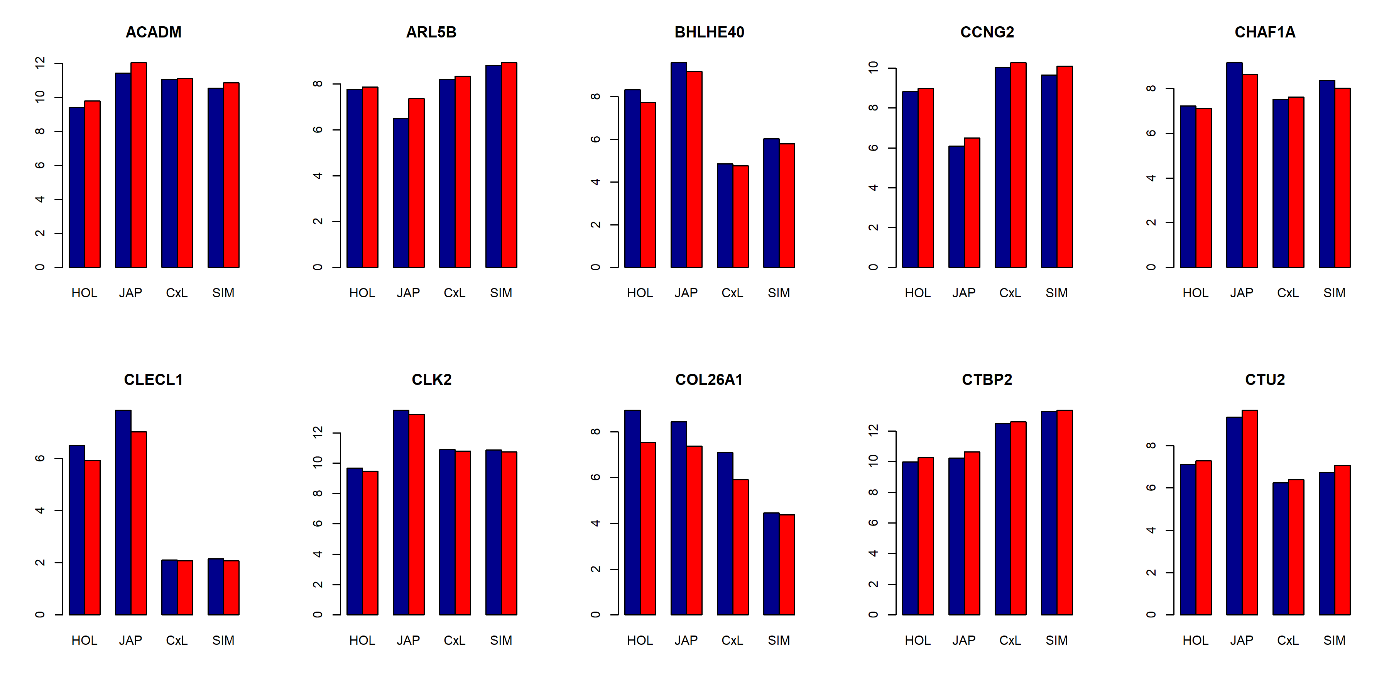
(A)


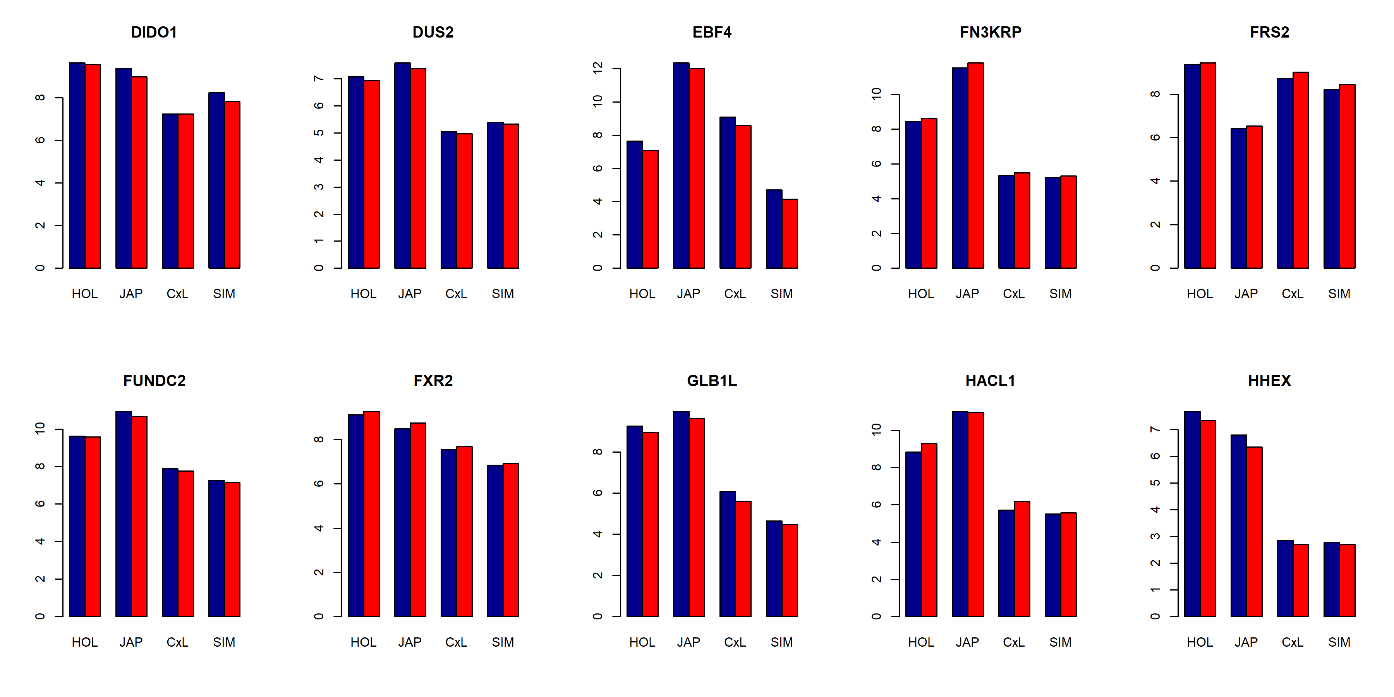


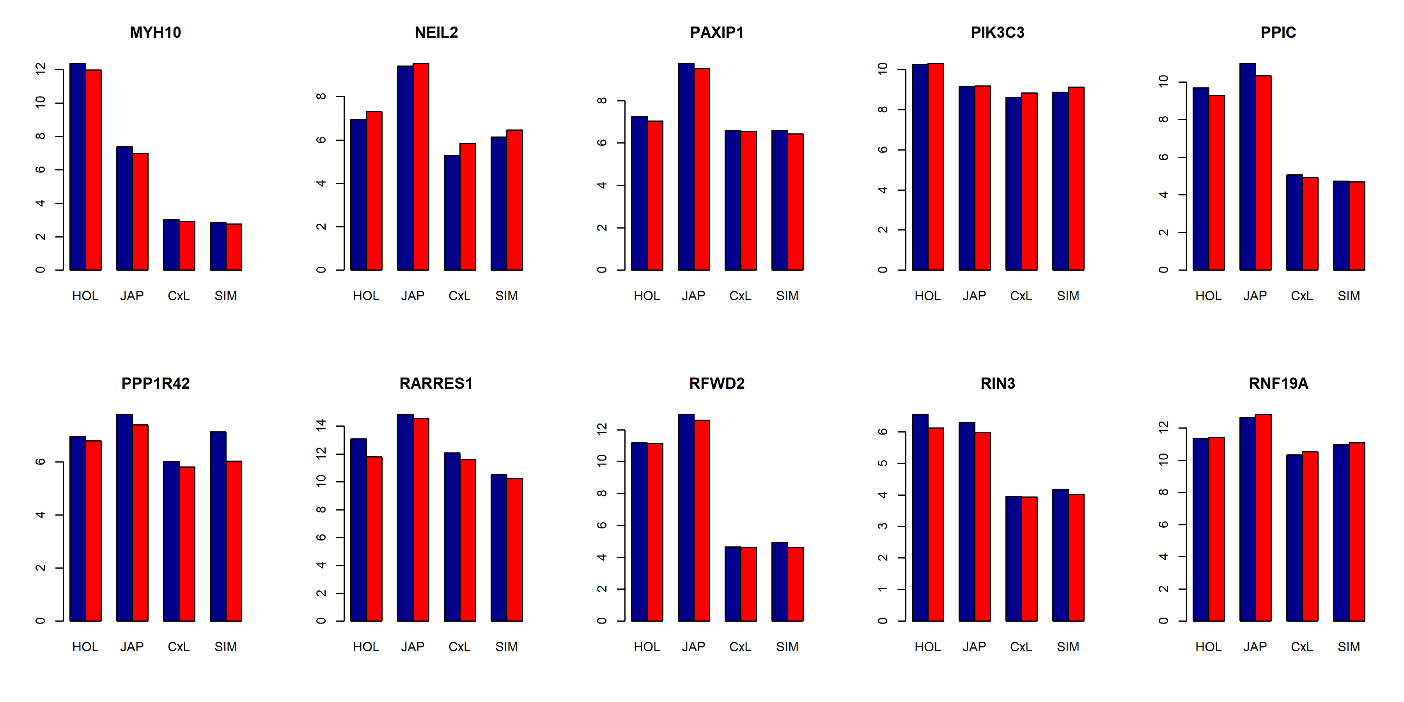


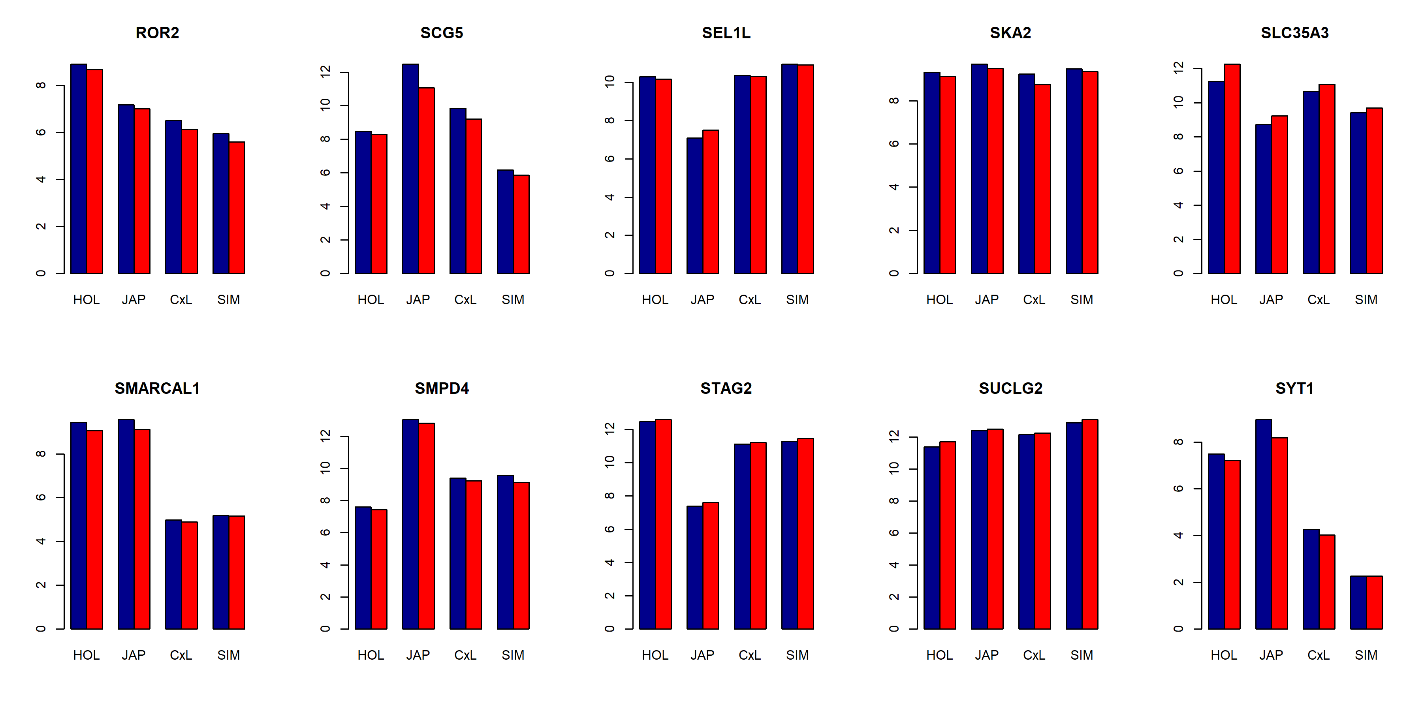


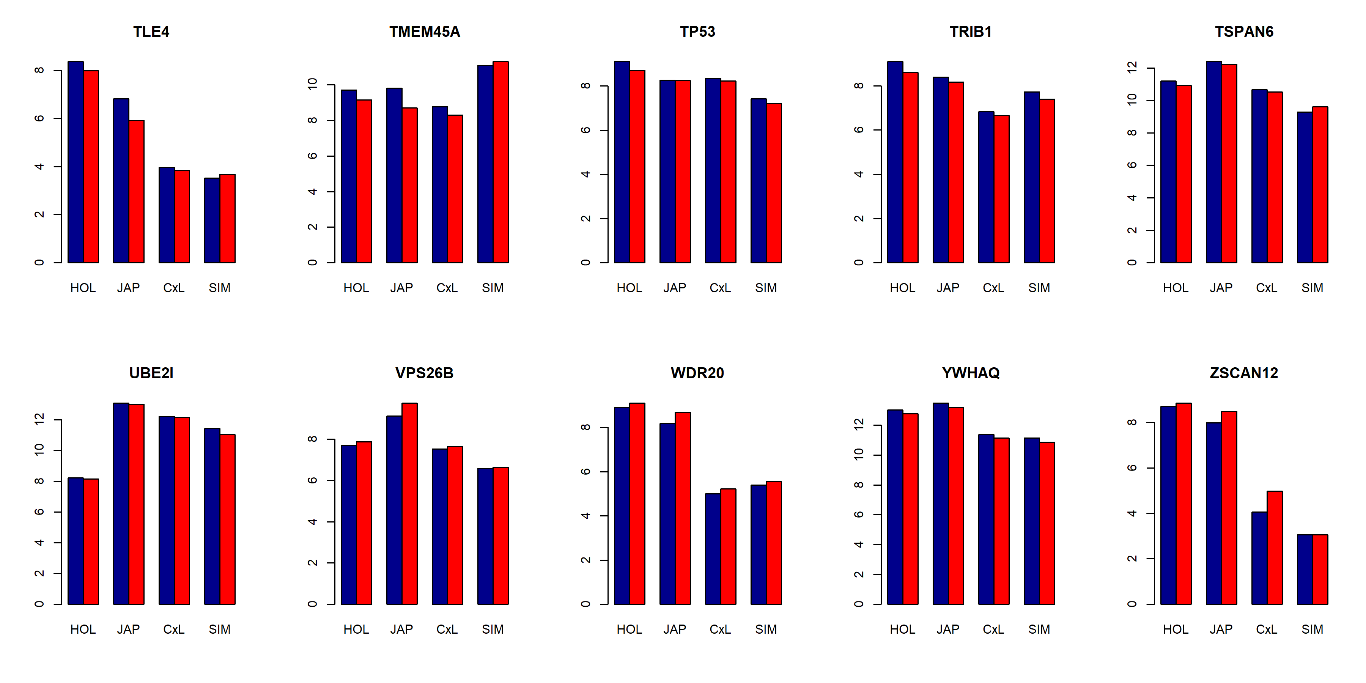


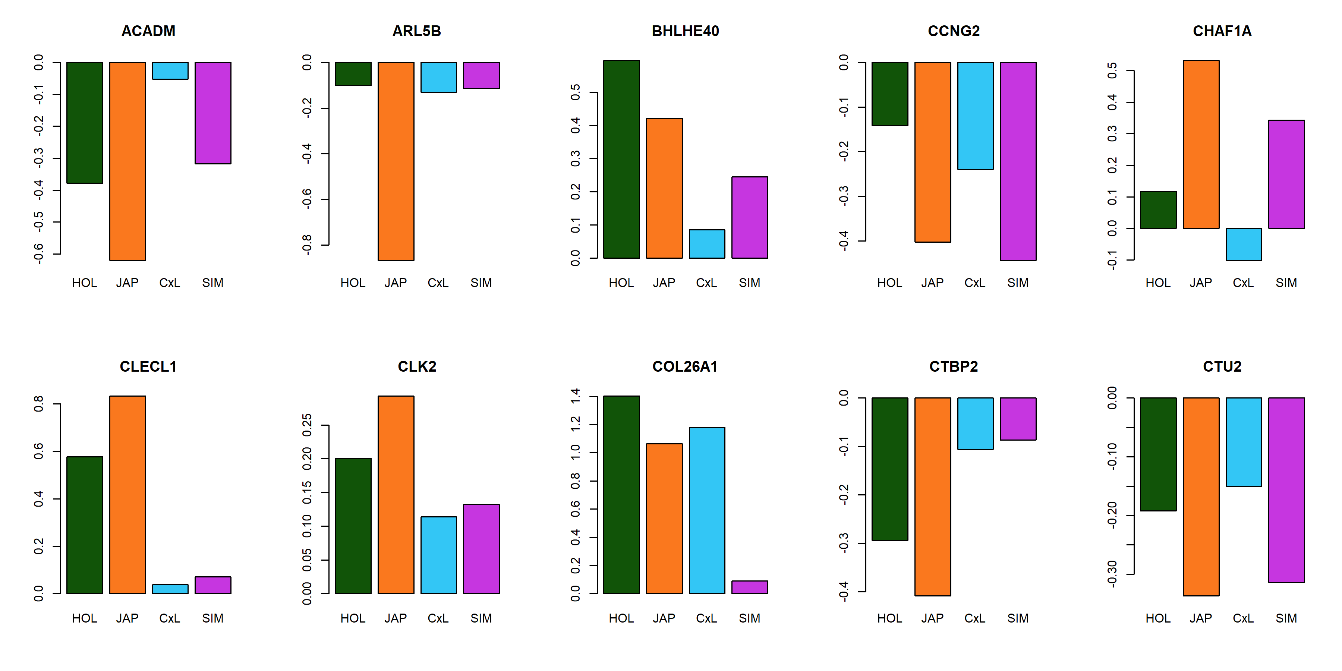
(B)


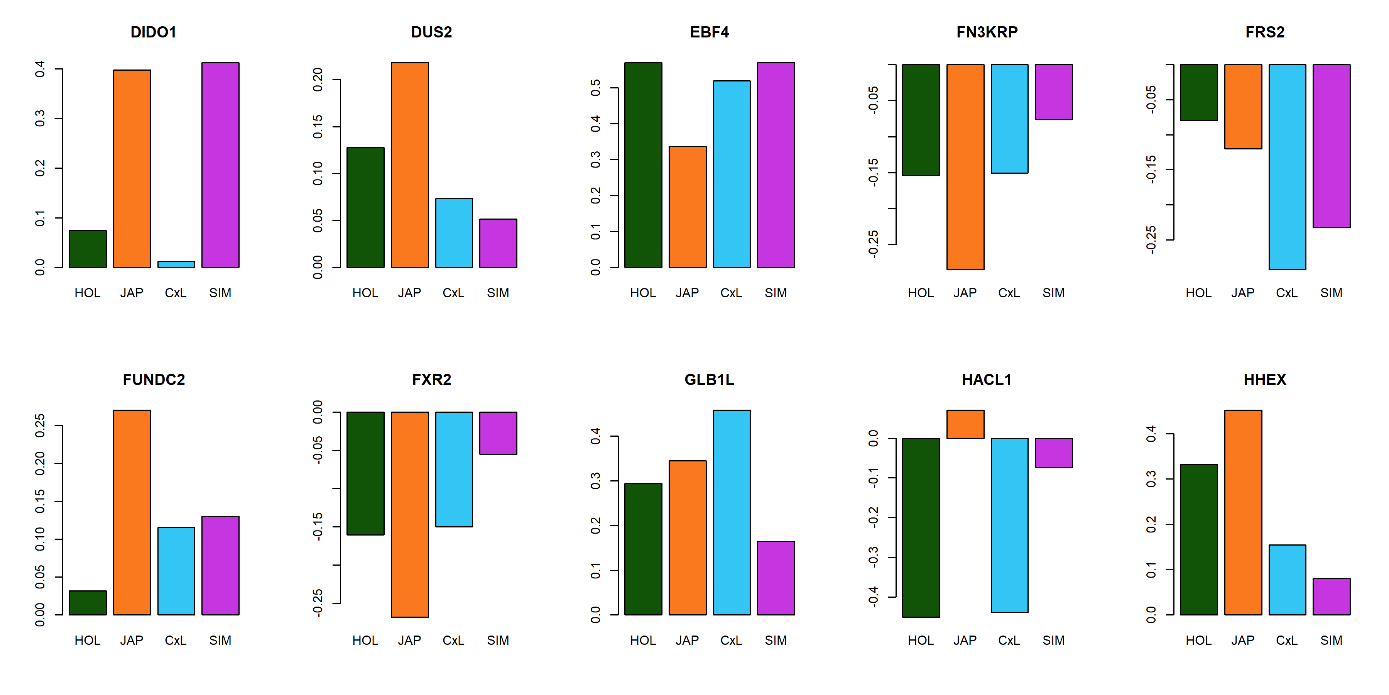


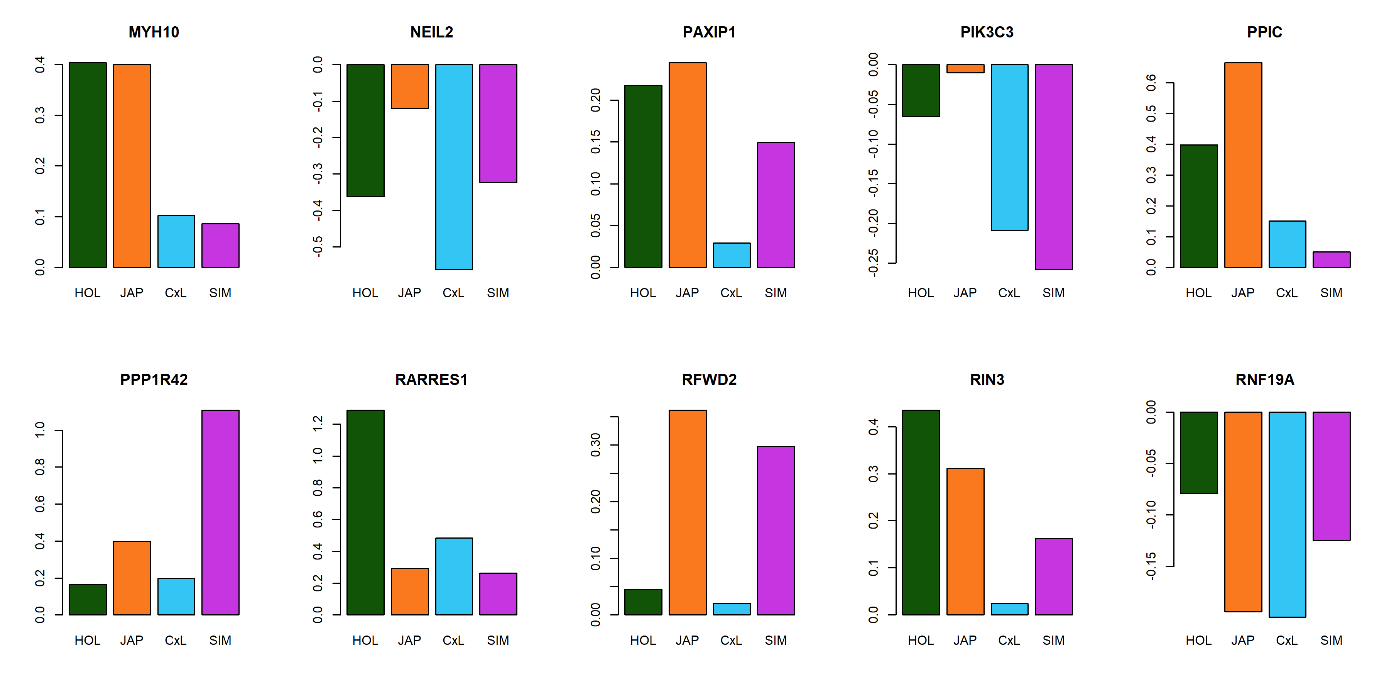


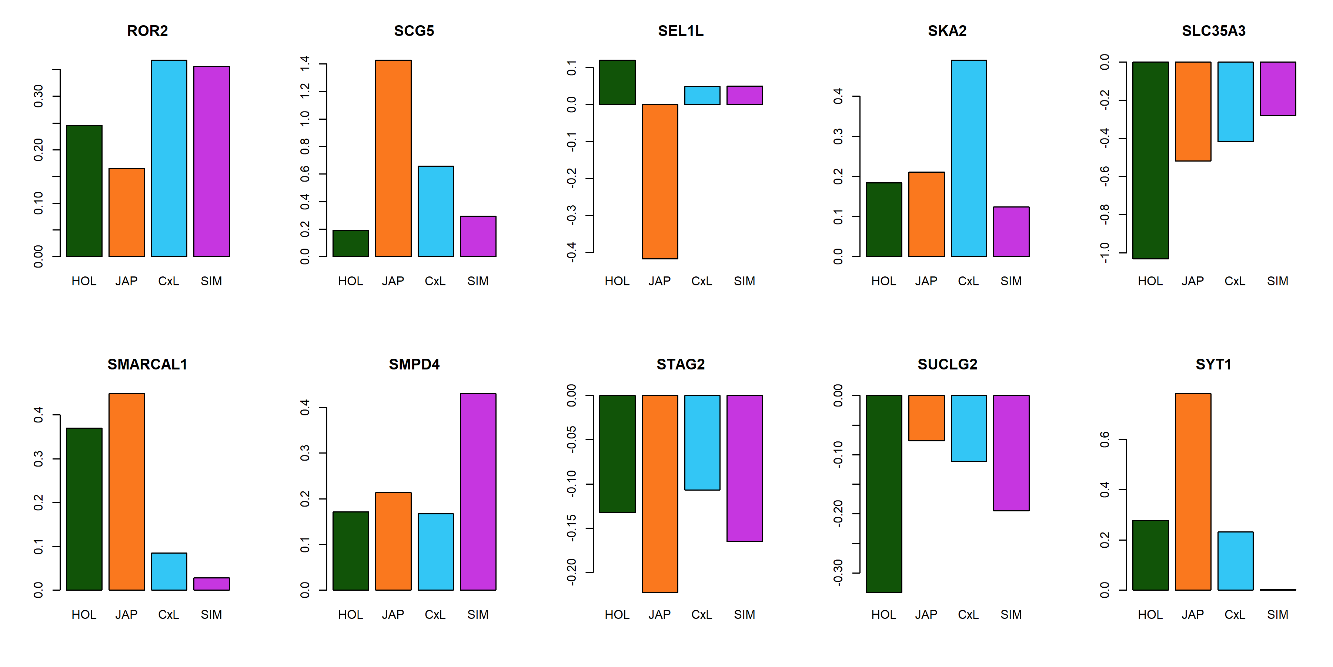


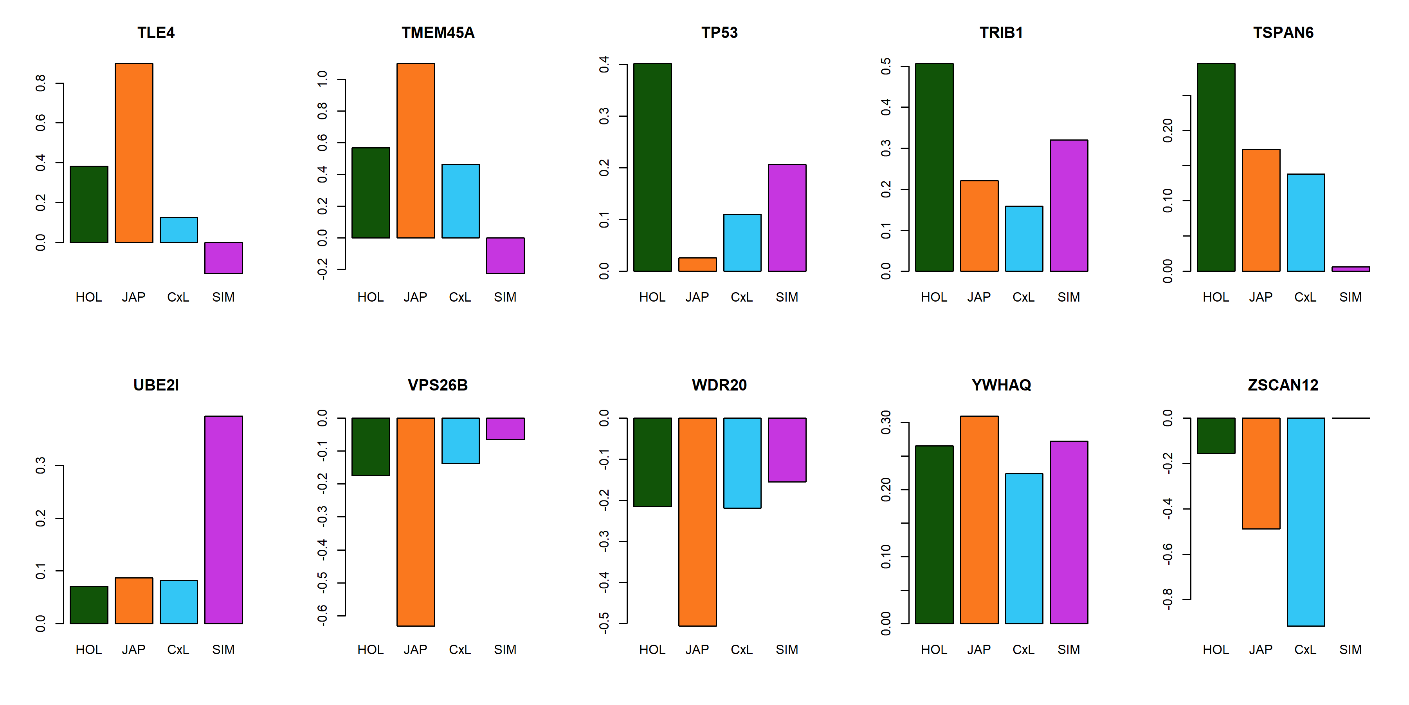

Supplement: Supplementary file 1 — Supplementary Information. [file 41598_2020_72988_MOESM1_ESM.docx]
